# Mast cell release of TNF-α indirectly contributes to recalling B cells in the colorectal cancer milieu through the CCL20/CCR6 axis

**DOI:** 10.3389/fimmu.2026.1725902

**Published:** 2026-04-21

**Authors:** Viviana Valeri, Francesca Mion, Silvia Tonon, Eleonora Martinis, Elena Jachetti, Roberta Sulsenti, Eleonora Capezzali, Serena Battista, Alessandro Mangogna, Laura Mariuzzi, Marco Fontanot, Beatrice Belmonte, Valeria Cancila, Claudio Tripodo, Marta Mozzon, Alessandro Uzzau, Barbara Frossi, Carlo Pucillo

**Affiliations:** 1 Immunology Section, Department of Medicine, University of Udine, Udine, Italy; 2 Molecular Immunology Unit, Department of Experimental Oncology, Fondazione IRCCS - Istituto Nazionale dei Tumori di Milano, Milan, Italy; 3 Pathology Department, Santa Maria della Misericordia Hospital, Udine, Italy; 4 Institute of Pathology, University Hospital of Udine, Azienda Sanitaria Universitaria Friuli Centrale (ASUFC), Udine, Italy; 5 Department of Medicine, University of Udine, Udine, Italy; 6 Department of Life Sciences, University of Trieste, Trieste, Italy; 7 Department of Health Science, Tumor Immunology Unit, Human Pathology Section, Palermo University School of Medicine, Palermo, Italy; 8 Advanced Pathology Laboratory, Istituto Fondazione di Oncologia Molecolare ETS (IFOM ETS)-The AIRC Institute of Molecular Oncology, Milan, Italy; 9 Department of Oncology and Hemato-Oncology, University of Milan, Milan, Italy; 10 General Surgery, University Hospital of Udine, ASUFC, Udine, Italy

**Keywords:** B cells, CCL20, colorectal cancer, lymph nodes, mast cells, TNF-α

## Abstract

**Introduction:**

In colorectal cancer (CRC), mast cells (MCs) modulate tumor and immune cell interactions, influencing patient prognosis, though their role remains not fully elucidated. Our group previously uncovered that in the intestine MCs provide support for the effector functions of B cells, both in physiology and inflammation.

**Methods:**

In this work we investigated the relationship between the activation of MCs in CRC and recruitment and accumulation of B cells in the tumor environment.

**Results:**

We observed infiltration of both B cells and MCs in the tumor tissue and uncovered accumulation of CCR6^+^ B cells in tumor lymph nodes (LNs), both in the mouse model and in human patients. Enhanced expression of the CCL20 chemokine, the ligand of CCR6, was observed in cancer tissue compared to the normal condition, along with an increased CCL20 gradient in mouse tumor-draining LNs. We proved that TNF-α released by activated-MCs was required to sustain CCL20 production from cancer cells *in vitro*.

**Discussion:**

The accumulation of CCR6^+^ B cells in CRC context may then rely on the crosstalk between MCs and CRC cells. Our findings suggest that B cell immunosurveillance may be indirectly promoted through the clinical modulation of TNF-α secretion at the early stages of colorectal tumorigenesis.

## 1 Introduction

Colorectal cancer (CRC) is the third most frequent malignancy and second cause of cancer death worldwide according to GLOBOCAN 2022 database (1). A close connection between inflammation and intestinal tumorigenesis is well-determined, although the mechanisms that link these conditions remain elusive. Increased cytokine and chemokine levels sustain inflammation and modulate cancer progression orchestrating interactions between cancer and immune cells (2). In the gut, an intimate relationship is established between mast cells (MCs) and B cells, both in homeostasis and inflammation, responsible for the regulation of IgA responses (3–6). MCs and B cells populate normal and inflamed intestine and infiltrate CRC tissue (3, 4, 7, 8). Whether their role is beneficial or detrimental in tumor progression is still unclear, since a multitude of environmental and genetic patient-specific factors can impact aspects of MCs’ and B cells’ phenotypes and activation (8, 9). Although the accumulation of MCs at the tumor edge has ambiguous prognostic significance (9), gene signature analyses suggest that among immune cells, MCs differ the most between normal and CRC tissue (10). Similarly, B cells may have prognostic value for patients since they can play essential roles in both anti-tumor and pro-tumor responses (e.g. production of killing antibodies, antigen presentation or induction of immunotolerance), and may become novel target of immune-related cancer therapy (11–14). While the stem cell factor (SCF)-driven MC recruitment and activation within tumors has been elucidated (15–17), the mechanisms leading to B cell migration into tumors are mostly unknown. In a preceding work, we showed that B cells accumulated in the tumor-draining lymph nodes (dLNs) of the Apc^Min/+^ and subcutaneous (s.c) CT26 tumor mouse models and we hypothesized that this was the result of the establishment of a lymph node (LN) environment enriched of chemokines that promote B cell recruitment (18). In this work we explored the relation between the activation of MCs in CRC and B cell recruitment/accumulation in the tumor milieu by establishing subcutaneous (s.c.) MC38 CRC cells growth in wild-type (Wt) mice and in Kit^W-sh^ MC-lacking mice or MC-reconstituted Kit^W-sh^ mice. We validated our data by analyzing B cells and MCs from human biopsies of CRC patients and investigated the potential effector functions of B cells isolated from LNs infiltrating the tumor in CRC patients. The bidirectional interaction between MCs and CRC cells was explored *in vitro* using co-cultures between bone marrow-derived MCs (BMMCs) and the MC38 cell line. We demonstrated that TNF-α, released by cancer-activated MCs, sustains CCL20 chemokine production by cancer cells. In the context of CRC, the accumulation of CCR6-expressing B cells may therefore depend on the crosstalk between MCs and CRC cells. This interaction, potentially conserved across different tumor contexts, may serve as basis for therapeutic strategies targeting MC activation to promote B cell-mediated antitumor responses.

## 2 Materials and methods

### 2.1 Animals, treatments, and isolation of tumor-bearing mouse tissues

Female C57BL/6-wild type (Wt) and C57BL/6-Kit^W-sh/W-sh^ (Kit^W-sh^) mice were purchased from Envigo or The Jackson Laboratory and maintained under specific pathogen-free conditions. 4- to 6-wks-old Kit^W-sh^ mice were reconstituted by intraperitoneal injection of 5x10^6^ *in vitro* differentiated bone marrow MCs (BMMCs). 4 weeks later, reconstituted Kit^W-sh^ mice, together with age- and sex-matched Wt and Kit^W-sh^ counterparts, were s.c. injected in the right flank with 2x10^5^ MC38 cells and sacrificed using cervical dislocation when tumor reached approximately 500 mm^3^. Once resected, tumors were mashed in serum-free culture medium supplemented with 0.25 mg/ml collagenase IV (Sigma-Aldrich) and 5 U/ml DNase (Roche), and incubated for 30-45min at 37 °C and 5% CO_2_. FBS was added to stop the reaction, and single-cell suspension was obtained using a cell strainer. Tumor cells were plated into multi-well plates to attain a 25-50% confluence for co-culture experiments. Tumor-draining LNs (right inguinal LNs) and not-draining LNs (left inguinal LNs) were collected separately. Spleen and LN cells were obtained by mechanical dissociation of organs through cell strainer. Red blood cells from spleens were lysed with ACK lysing buffer. Samples from TNF-α^-/-^ mice were kindly provided by Prof. Kollias, BSRC “Alexander Fleming”, Vari, Greece. Animal housing and experimentation were performed in adherence to ARRIVE Guidelines and conducted in compliance with Italian legislation (D.lgs 26/2014). Animal use was minimized to ensure statistically valid outcomes. *In vivo* experiments, were approved by the Italian Ministry of Health (authorization numbers 634/2015-PR and 1085/2015-PR).

### 2.2 Mouse MC38 and primary cells culture conditions

The MC38 colon adenocarcinoma cell line, kindly provided by Prof. Bronte, University of Verona, Italy, was cultured in complete DMEM. B cell isolation kit (Miltenyi) was used to isolate B lymphocytes from splenocytes for co-culture experiments with BMMCs. BMMCs, obtained by *in vitro* differentiation of progenitors from femurs and tibiae of Wt and TNF-α *^-/-^* mice, were cultured in the presence of 20 ng/ml IL-3 (PeproTech) as previously described (4). For co-culture experiments, cells were cultured in complete RPMI. Where indicated, IL-33 (PeptoTech) or TNF-α blocking mAb (MP6-XT22, eBioscience) was added to the cell culture medium.

### 2.3 Human colon biopsies and cells

2 g of CRC tissue and 20 cm away adjacent normal colon, cleared of blood cells and adipose tissue, were reduced into 2 mm pieces, moved in DMEM/F12 medium (supplemented with 100 U/ml penicillin, 100 mg/ml streptomycin, 2 mM L-glutamine, 20 mM Hepes, 100 mg/ml Primocin™ (InvivoGen), 50μg/ml Liberase (Merck) and 10 µM Y-27632 dihydrochloride (ProdottiGianni) and maintained for 1 hour at 37 °C under gentle shaking to allow tissue digestion. The digested suspension or cleaned LNs from CRC and adjacent normal tissue were filtered through a cell strainer to obtain a single-cell suspension. Where indicated, LN cells were cultured in complete RPMI medium in the presence of anti-CD40 (Sony) plus IL-21 (PeproTech) or IL-2 (PeproTech) plus CpG (Merck) for 7 days or with the combination of CpG, PMA, Ionomycin (Merck) and Brefeldin A (ThermoFisher) for 5h. Biopsies of CRC and adjacent normal tissue, collected since April 2024, were provided by Dipartimento Chirurgico S. Maria della Misericordia, Azienda Sanitaria Universitaria Friuli Centrale. The study was approved by the appropriate local institutional ethic committee (Comitato Etico Unico Regionale (CEUR FVG); ID of the study 17444, prot. 0055270-26.04.2024) and conducted in accordance with the Declaration of Helsinki; written informed consent was obtained from all participants.

### 2.4 Flow cytometry

Whenever possible, flow cytometry data acquisition and analysis were performed following Cossarizza et al. (19). Cell suspensions were incubated for 30 minutes on ice with fluorochrome-labeled Abs and, whenever possible, Live/Dead viability marker (Invitrogen) in PBS 0.5% BSA. For intracellular staining, cells were first stained for surface antigens, then fixed and permeabilized with the Cytofix/Cytoperm kit (BD Biosciences). Cells were resuspended in PBS 2% FBS and acquired on FACSCalibur (Becton Dickinson) or Attune NxT flow cytometer (ThermoFisher). Analyses were performed with FlowJo software. Staining reagents are listed in **Supplementary Table 1**.

### 2.5 RNA extraction and RT-qPCR

RNA was extracted using EUROGOLD TriFast (Euroclone) according to manufacturer’s instructions and quantified with NanoDropTM (ThermoFisher). One microgram of RNA was reverse transcribed with the SensiFAST™ cDNA Synthesis Kit (Bioline). RT-qPCR analyses were performed with SYBR Green chemistry (iQ™ SYBR Green Super Mix, BioRad) using the BioRad CFX96real-time PCR detection systems. Target gene expression was quantified using *Gapdh* or *Beta-2 microglobulin* (*B2M*) as normalizer genes for mouse and human cDNA samples respectively. Primers used for RT-qPCR (Merck) are listed in **Supplementary Table 2**.

### 2.6 Quantification of secreted mediators

Cell supernatants were analyzed using ELISA commercial kits for the detection of mouse TNF-α, IL-6, IL-13, CCL20 and human serum CCL20 (ThermoFisher) or mouse SCF (RayBiotech) according to manufacturer’s instructions.

### 2.7 Histology and immunohistochemistry

#### 2.7.1 Mouse samples

Four-micrometer–thick tissue sections were deparaffinized, rehydrated, and unmasked using Novocastra Epitope Retrieval Solutions at pH9 (Leica Biosystems) at 98 °C for 30 minutes. The sections were then brought to RT and washed in PBS. After neutralization of the endogenous peroxidase with 3% H_2_O and Fc blocking with 0.4% casein in PBS (Leica Biosystems), the sections were incubated with rat anti-mouse CD45R (RA3-6B2 1/10 pH9, BD Pharmigen), anti-Mast Cell Tryptase (EPR 8476, dilution 1:500, pH6, ab134932), anti-CCL20 (dilution 1:500, pH9, ab9829), anti-CD45 (dilution 1:500, pH9, ab10558) and anti-CD31 (1:50, pH9, ab28364) primary antibodies. Immunohistochemistry (IHC) staining was developed using the IgG (H&L)-specific secondary antibodies (Life Technologies, 1:500). Double IHC staining was performed using the SignalStain Boost IHC Detection Reagent alkaline phosphatase-conjugated (anti-rabbit; Cell Signaling Technology) and the IgG (H&L)-specific secondary antibodies (Life Technologies, 1:500). DAB (3,3′-Diaminobenzidine, Novocastra) and Vulcan Fast Red were used as substrate chromogens. For multiple-marker immunofluorescence staining, sections underwent sequential rounds of single-marker immunostaining with the binding of primary antibodies revealed using specific secondary antibodies conjugated with different fluorophores. DAPI (4′,6-diamidin-2-fenilindolo) was utilized to counterstain the nuclei. Slides were analyzed under a Zeiss Axioscope A1 microscope microphotographs were collected using a Zeiss Axiocam 503 Color digital camera with the Zen 2.0 Software (Zeiss). Quantitative IHC data was calculated by counting the number of CD45R^+^ and Tryptase^+^ cells in five fields. Quantitative analyses of CCL20 staining was performed by calculating the average percentage of positive cells in five non-overlapping fields at medium-power magnification (x200) using the HALO image analysis software (v3.2.1851.229, Indica Labs) and the output was expressed as the “percentage of positive cells”.

#### 2.7.2 Human samples

Formalin-fixed, paraffin-embedded tumor and LN tissue sections were stained with primary antibodies against CD20 (M0755, Dako), Tryptase (M7052, Dako) and CCL20 (PA5-47517, Thermofisher). The localization of B lymphocytes, MCs, and the chemokine CCL20 was assessed by light microscopy and categorized as intratumoral, peritumoral, within peritumoral lymphoid aggregates, and in healthy mucosa distant from the neoplastic lesion. The IHC results were independently evaluated in a blinded manner by three experienced pathologists (L.M., S.B., and A.M.). The IHC score was assigned on a scale from 0 to 3 according to both the percentage of positive cells and the intensity of staining. Staining for B cells and MCs was classified as follows: absent (Grade 0), mild or moderate focal infiltrate or mild multifocal infiltrate (Grade 1), marked focal, moderate or marked multifocal, or mild diffuse infiltrate (Grade 2), and moderate or marked diffuse infiltrate (Grade 3). For the IHC assessment of the chemokine CCL20, the same scoring system was adopted, but the term infiltrate was replaced with expression. Tissue sections were examined and digital images acquired using standard light microscopy with the Leica Aperio AT2 DX System (Leica Biosystems, Vista, CA, USA). Details regarding antibodies used are listed in **Supplementary Table 3**.

### 2.8 Statistics

Mann-Witney test, one-sample t test, Wilcoxon matched-pairs test, Kruskal-Wallis analysis with uncorrected Dunn’s test or Friedman test were performed with GraphPad Prism 9 Software. P values <0.05 were considered statistically significant.

## 3 Results

### 3.1 B cells and MCs are expanded in tumor-draining LNs of MC38 tumor-bearing mice where an increased gradient of CCL20 chemokine is established

**Figure 1:**
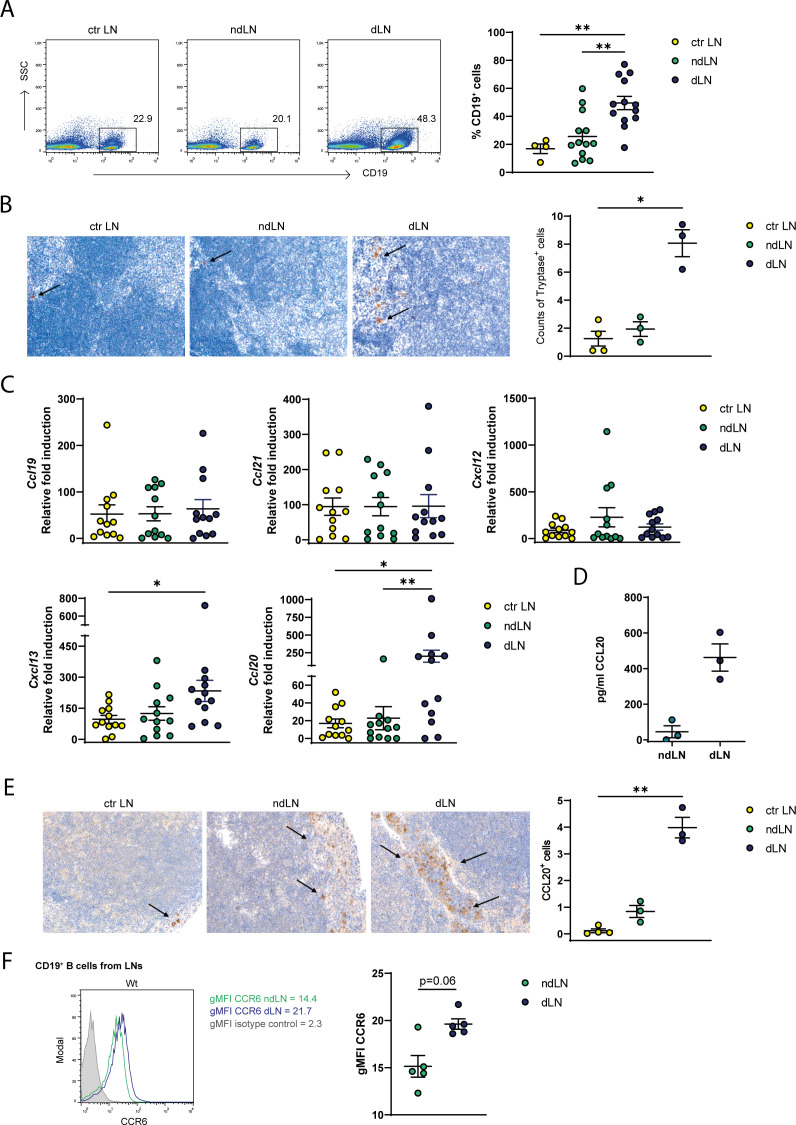
B cells and MCs are expanded in tumor-draining LNs of MC38 tumor mice where an increased gradient of CCL20 is established. **(A)** Percentages of CD19^+^ B cells were analyzed by flow cytometry in cell suspensions obtained from control (ctr LN) or draining (dLN) and non-draining (ndLN) LNs of MC38 tumor-bearing mice. **(B)** Representative analysis of Tryptase immunostaining of LN sections from control, nd and dLNs. Counts of Tryptase^+^ are provided in the scatter plot where each symbol is the mean count of 5 fields (x200). Each symbol depicts an individual mouse among the groups analyzed. **(C)** The relative expression of *Ccl19*, *Ccl21*, *Cxcl12*, *Cxcl13* and *Ccl20*, normalized to the housekeeping gene *G3pdh*, was analyzed by qPCR in the total cell population of ctr LN, ndLN and dLNs isolated from control and tumor-bearing mice. For each chemokine, the control sample with the lowest expression was used as control and set to 1. **(D)** CCL20 chemokine was quantified by ELISA in supernatants derived from ndLN and dLN of MC38 tumor-bearing mice sampled and placed *ex vivo* in culture medium for 24h. Each symbol depicts an individual mouse. **(E)** Representative images of CCL20 immunostaining of LNs from control, nd and dLNs. Counts of CCL20^+^ cells are provided in the scatter plot where each symbol is the mean count of 5 fields (x200). Each symbol depicts an individual mouse among the groups analyzed. **(F)** CCR6 expression, showed as geometric Mean Fluorescence Intensity (gMFI), was determined by flow cytometry in CD19^+^ B cells obtained from dLNs and ndLNs of MC38 tumor-bearing mice. On the left, a representative histogram plot is shown. Means ± SEM are shown in the graphs. Kruskal-Wallis analysis with uncorrected Dunn’s test was performed for multiple comparisons; Wilcoxon matched-pair test was used in panels **(C, D)** *p<0.05, **p<0.01.

Our study began by exploring how tumor development affects the distribution of B cells in peripheral lymphoid districts. Hence, we determined B cell amounts in the spleen, peritoneal cavity and LNs in mice bearing s.c. MC38-derived tumors compared to control mice through flow cytometry. We observed that B cell frequencies were unchanged in anatomical sites distal to the tumor (spleen and peritoneal cavity, **Supplementary Figure 1**). Conversely, B cells accumulated in tumor draining lymph nodes (dLNs) with respect to not-draining LNs (ndLNs) and control mice pooled LNs (**Figure 1A**), in contrast we observed a decreased proportion of CD4^+^ T cells in the dLNs (**Supplementary Figure 2**). We also detected MCs, which are rarely present in LNs from control mice and in ndLNs, but accumulated in the dLNs of tumor−bearing mice (**Figure 1B**). To identify possible mediators for B cell recruitment, we analyzed the expression of B-cell related chemokines through qPCR in dLNs *versus* ndLNs. *Ccl19*, *Ccl21* and *Cxcl12* chemokine expression was not modulated between control, ndLNs and dLNs while the expression of *Cxcl13* and *Ccl20* resulted increased in dLNs compared to control LNs (**Figure 1C**). An increased production and release of CCL20 from *ex vivo* harvested LNs left for 24h in culture medium was confirmed by ELISA (**Figure 1D**) and through immunohistochemical evaluation (**Figure 1E**). Moreover, we performed double−immunofluorescence staining for CCL20 together with EpCAM, CD31, or CD45 to determine the main cell populations producing CCL20 in tumor dLNs, marking MC38 tumor cells, endothelial cells, or immune cells, respectively. No identifiable EpCAM^+^ epithelial cells were observed within dLNs (data not shown); in contrast, subsets of both CD31^+^ endothelial cells and CD45^+^ immune cells exhibited detectable co−staining with CCL20 (**Supplementary Figure 3**). We further assessed CCR6 expression—the only known receptor for the CCL20 chemokine on B cells—in B cells accumulated in ndLNs and dLNs. The results showed a trend towards upregulation in the dLNs (**Figure 1F**). Altogether our data suggest a CCL20/CCR6 driven B cell accumulation in tumor-dLNs.

### 3.2 The presence of MCs promotes the accumulation of B cells within the tumor

**Figure 2:**
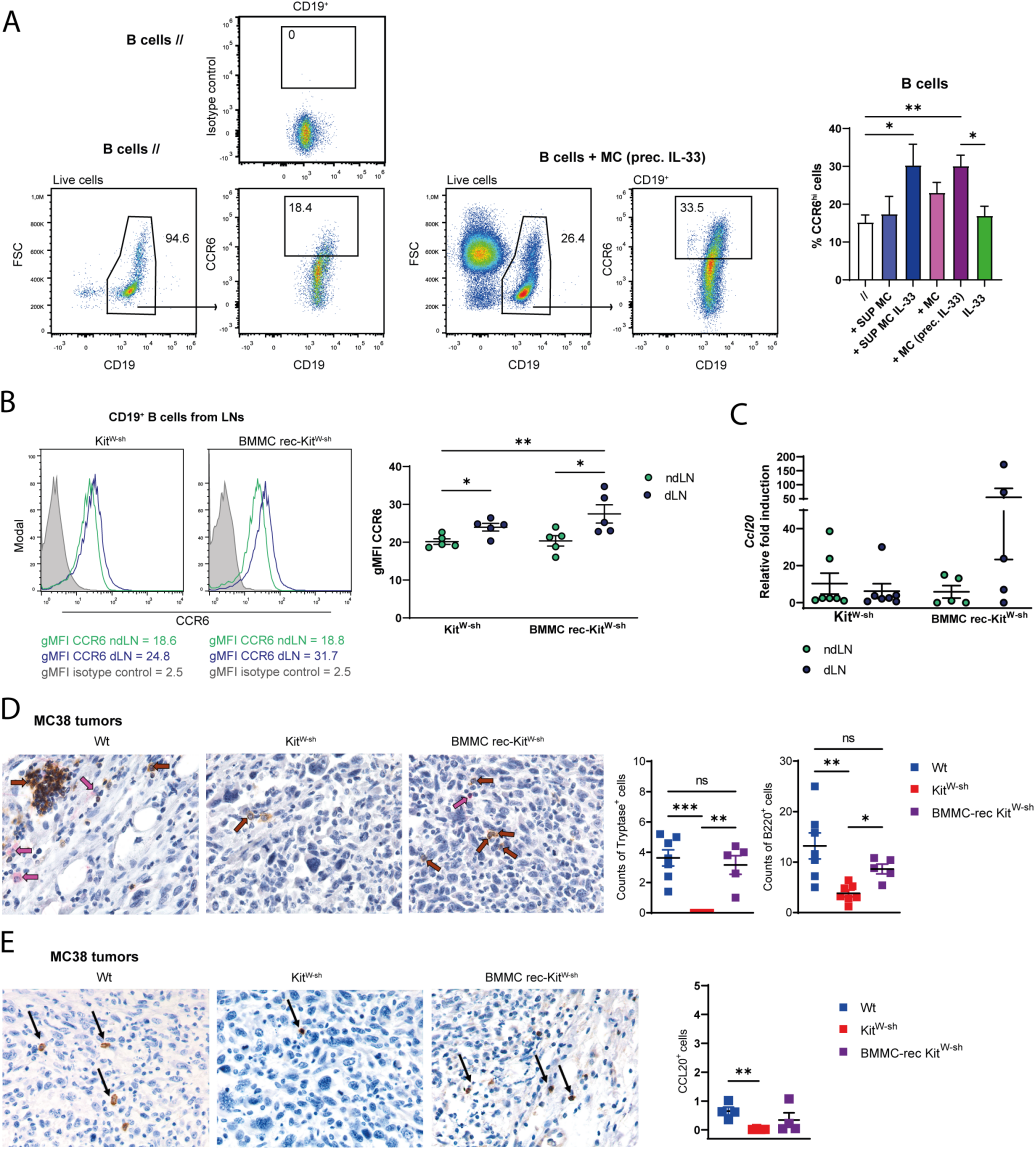
The presence of MCs promotes the accumulation of B cells within the mouse tumors. **(A)** Percentages of CCR6^hi^ B cells were determined after culturing splenic purified B cells from control mice for 48h in the following conditions: alone resting (//), in the presence of 24h supernatant derived from resting or 50 ng/ml IL-33 stimulated BMMCs (+ SUP MC and + SUP MC IL-33 respectively), or in co-culture with resting (+ MC) or 50 ng/ml IL-33 pre-stimulated BMMCs (+ MC (prec. IL-33)), or with 50 ng/ml IL-33. **(B)** CCR6 expression, showed as gMFI, was determined by flow cytometry in CD19^+^ B cells obtained from dLNs and ndLNs of Kit^W-sh^ and BMMC rec-Kit^W-sh^ MC38 tumor-bearing mice. On the left, representative histogram plots are shown. **(C)** Relative expression of *Ccl20*, normalized to the housekeeping gene *G3pdh*, was analyzed in the total cellular population of LNs isolated from Kit^W-sh^ and BMMC rec-Kit^W-sh^ tumor mice by qPCR. For each mouse setting, the ndLN sample with the lowest expression within each group of mice was used as control and set to one. **(D)** Representative analysis of Tryptase (pink, indicated by arrows) and B220 (brown, indicated by arrows) immunostaining of MC38 tumor sections from Wt, Kit^W-sh^ and BMMC rec-Kit^W-sh^. Counts of Tryptase^+^ and B220^+^ cells are provided in the scatter plots where each symbol is the mean count of 5 fields (x400). Each symbol depicts an individual mouse among the groups analyzed. **(E)** Representative images of CCL20 immunostaining of MC38 tumors from Wt, Kit^W-sh^ and BMMC rec-Kit^W-sh^ mice. Counts of CCL20^+^ cells are provided in the scatter plots where each symbol is the mean count of 5 fields (x200). Each symbol depicts an individual mouse among the groups analyzed. Means ± SEM are shown in **(B–E)**. Means +SEM are shown in **(A)**. Kruskal-Wallis analysis with uncorrected Dunn’s test was performed for multiple comparisons. *p<0.05, **p<0.01, ***p<0.001. ns, not statistically significant.

We found that MCs and B cells accumulate in the dLNs of MC38−bearing mice. It is also well documented that activated MCs are well-established modulators of B cell surface molecules (3, 4, 20). Thus, we established *in vitro* BMMCs-B cells co-culture experiments to evaluate whether MCs could modulate CCR6 expression on B cells. Since we recently published that MCs, in CRC, are critically activated through the IL-33/ST2 axis (21), we cultured resting B cells for 48h with either untreated or IL-33 pre-stimulated MCs, or their conditioned medium (CM). We observed an increase in the percentage of CCR6^hi^ B lymphocytes when cells were cultured with the CM derived from IL-33-stimulated BMMCs or IL-33-preconditioned BMMCs (**Figure 2A**).

MC38 tumor growth was next induced in MC-deficient Kit^W-sh^ mice and BMMC-reconstituted Kit^W-sh^ mice and we assessed CCR6 expression on LN B cells as performed in **Figure 1F**. Although CCR6 upregulation was observed in B cells accumulating in dLNs compared to ndLNs across both mouse groups, CCR6 expression was highest in Kit^W-sh^ tumor mice repopulated with BMMCs prior to tumor injection (**Figure 2B**).

We further compared *Ccl20* chemokine expression in ndLNs *versus* dLNs and no observable changes were found in the Kit^W-sh^ mouse system. In BMMC-reconstituted Kit^W-sh^ tumor-bearing mice, instead, we observed a trend approaching the condition seen in Wt tumor mice (**Figures 1C**, **2C**).

Since B cells accumulated in dLNs compared to ndLNs in both mouse backgrounds analyzed (**Supplementary Figure 4**), we may assume a B cell accumulation independent of CCL20 in the absence of MCs. The ability of B cells to migrate more efficiently toward a CCL20 gradient could instead contribute to their enhanced accumulation within the tumor. To investigate the dynamics of MC and B cell infiltration in the tumor tissue, we performed double immunohistochemistry to simultaneously detect MCs and B cells within the MC38 s.c. tumors. MCs, identified as Tryptase^+^ cells, absent in tumors from Kit^W-sh^ mice, populated the tumors of BMMC reconstituted Kit^W-sh^ mice at levels comparable to those observed in Wt tumor-mice (**Figure 2D**, left plot). Counts of B220^+^ B cells were also assessed: the analysis showed reduced infiltration of B cells within the tumor grown in the absence of MCs suggesting a plausible interdependency of distribution between the two cell types (**Figure 2D**, right plot). To determine CCL20 levels in mouse tumors, we performed immunohistochemistry staining for CCL20 on tumor sections from Wt, Kit^W-sh^ and BMMC-reconstituted Kit^W-sh^ mice. CCL20 positivity reflected MC infiltration within the tumors: it was reduced in Kit^W-sh^ mice, that lack MCs, and displayed a trend toward recovery in BMMC-rec Kit^Wsh^ (**Figure 2E**). These results support an association between tumor-infiltrating MCs and intratumoral CCL20 expression.

Our results, together, suggest that tumor infiltrating-MCs may promote the recruitment/accumulation of B cells within the tumor through the CCR6/CCL20 axis in a contact-mediated or independent fashion and our data clearly show that MCs sustain the presence of B cells within CRC.

### 3.3 MCs and CCR6^hi^ B cells populate the tumor tissue in CRC patients

**Figure 3:**
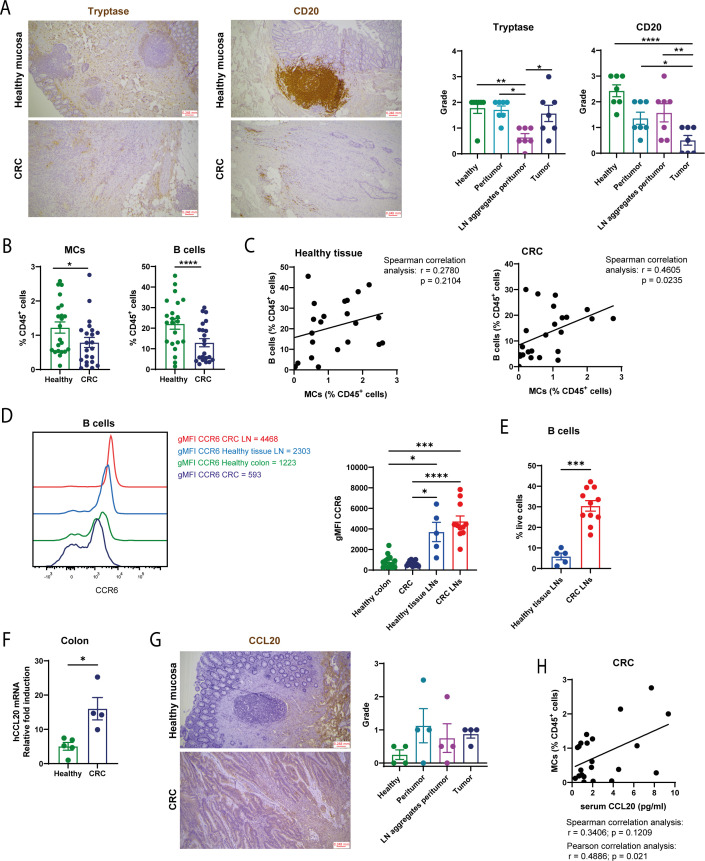
MCs and CCR6^hi^ B cells populate the tumor tissue of CRC human patients. **(A)** Evaluation of MC and B cell distribution in healthy, peritumor, peritumor LN aggregates and tumor areas was assessed by using Tryptase and CD20 markers in immunohistochemical analysis. Representative histological images are shown on the left. Histological grade is showed in the plots on the right. **(B)** Percentages of cKit^+^FcϵRI^+^ (MCs) and CD19^+^ B cells were determined through flow cytometry analysis performed on digested cell suspension of both healthy colon and CRC tissue and shown as percentages of CD45^+^ cells. **(C)** Spearman correlation analysis has been conducted for B cell and MC percentages for both healthy colon and CRC tissue; r and p values are indicated in the plot. **(D)** gMFI of CCR6 was determined by flow cytometry on CD19^+^ B cells from healthy or tumoral colon LNs and from healthy or CRC LN B cells. **(E)** Percentages of B cells were determined in healthy and tumor LNs within total live cells. **(F)** Relative expression of Ccl20, normalized to the housekeeping gene *B2M*, was analyzed in the total cellular population of healthy and tumoral digested colon tissues. **(G)** Evaluation of Ccl20 distribution in healthy, peritumor and tumor areas and in peritumor lymphoid aggregates was assessed by using immunohistochemical analysis. Histological grade is showed in the plot. **(H)** Correlation analysis has been conducted between CCL20 serum levels (determined through ELISA) and MCs density within cancer tissue; r and p values are indicated in the plot. Each symbol represents an individual patient. Means ± SEM are shown in **(A, B, D-G)**. Friedman test has been used for multiple comparisons in **(A, G)**. Wilcoxon matched-pairs test has been used in **(B)** Kruskal-Wallis analysis with uncorrected Dunn’s test was performed in **(D)** Mann-Whitney test has been used in E,F. *p<0.05, **p<0.01, ***p<0.001, ****p<0.0001.

We further analyzed freshly isolated biopsies of stages 2–3 CRC patients who had not undergone neoadjuvant therapy (additional patient information is provided in **Supplementary Table 4**) (22). Human MCs and B cells infiltrating the colon were firstly identified by immunohistochemistry utilizing Tryptase and CD20 markers to detect MCs and B cells, respectively. Corresponding histological immune infiltration score was evaluated. Presence of MCs was determined in normal lamina propria (called “Healthy” in the figure plots), in peritumoral space and within the tumor (often in dense foci). Rare MCs were observed close to the peritumoral lymphoid aggregates (**Figure 3A**). B lymphocytes were detected in normal colon, concerning the tumor area they were mostly localized in lymphoid aggregates in the peritumoral space, and less observed within the tumor (**Figure 3A**). We further assessed through flow cytometry immune cells infiltrating the CRC tissue and the adjacent normal mucosa and determined fractions of MCs (Fɛ̝RI^+^c-Kit^+^ cells) and CD19^+^ B cells in both healthy and tumoral colon (representative gating plots shown in **Supplementary Figure 5**). Percentages of MCs and B cells resulted decreased in the tumor tissue (**Figure 3B**), as already shown before for both cell populations (13, 23) and, especially for B cells, as confirmed by histology shown in **Figure 3A**. To dissect the B cell-MC interdependency, we analyzed their relative proportions. Regression analysis between B cells and MCs showed no correlation for the healthy condition, whereas a significant positive correlation was found in tumor tissue (**Figure 3C**). This result suggests a connection between the amounts of B cells and MCs infiltrating the tumoral intestine, in line with what emerged in the mouse model (**Figure 2D**). To further support the specificity of the association between MCs and B cells within tumor tissue, we assessed the distribution of CD4^+^ T cells in healthy mucosa and tumor samples, as well as the correlation between MCs and CD4^+^ T cells. Our data showed no significant differences in CD4^+^ T-cell numbers between the two conditions. Moreover, unlike the strong correlation observed between B cells and MCs, no statistically significant correlation was found between MCs and CD4^+^ T cells (**Supplementary Figures 6A, B**).

In order to validate the B cell accumulation in tumor LNs determined in the mouse setting (**Figures 1**, **2**), we isolated LNs enclosed within our biopsies in both the tumor mass and healthy colon. Histological analysis showed a normal distribution of B cells within follicles (**Supplementary Figure 7A**), while MCs, even though poorly represented in the lymphoid tissue, were more abundant in proximity to a metastatic LN (**Supplementary Figure 7A**). Moreover, immunohistochemical analysis performed on LN samples showed that CCL20 expression was predominantly localized to tumor cells within metastatic deposits, whereas the surrounding lymphoid tissue was largely negative (**Supplementary Figure 7B**). Hence, in CRC LNs, metastatic tumor cells appeared to be the main source of CCL20 levels, where heterogeneous but generally mirrored the expression of CCL20 was detected in the patient’s corresponding primary tumor tissue.

In mice, we hypothesized that B cells were recruited to the tumor environment driven by a CCL20 gradient. Analyzing the expression of CCR6, we observed that B cells compartmentalized within both control and CRC LNs displayed a higher CCR6 phenotype compared to B cells interspersed within the colon tissue (**Figure 3D**). Moreover, in line with the findings from the mouse model in **Figure 1A**, an accumulation of CD19^+^ cells in CRC LNs compared to healthy colon LNs was obtained (**Figure 3E**, **Supplementary Figure 8A** for the gating strategy), resulting in an overall accumulation of CCR6^+^ B cells (**Supplementary Figure 9**). Differently from B cells, CCR6 expression is not uniform on the entire CD4^+^ T cell compartment in both CRC tissue and LNs (**Supplementary Figures 6C-E**).

We hypothesized that, in CRC patients, CCL20 drives B cell infiltration to the TME and promotes their accumulation within lymphoid structures. To evaluate this, we analyzed the CCL20 mRNA levels through qPCR from cell lysates obtained from fresh biopsies. Our results confirmed higher expression of this chemokine in cancer tissue compared to healthy colon (**Figure 3F**). Furthermore, to validate qPCR analysis we assessed CCL20 expression from healthy tissue, peritumor and tumor area, and within peritumoral LNs through immunohistochemistry observing denser staining pattern in peritumoral and tumor zones compared to the healthy area (**Figure 3G**). These last two analyses support the hypothesis of a CCL20 enriched cancer milieu favoring the infiltration of CCR6^+^ B lymphocytes that further accumulate within tumor LNs. We finally quantified CCL20 in the serum of our patients through ELISA and correlated, for each patient, its amount with MC abundance in CRC intestine. A positive correlation between the two parameters was found (**Figure 3H**), suggesting that higher MC frequency within the tumor favors CCL20 production.

To recapitulate, in CRC biopsies we uncovered an accumulation of CCR6^+^ B cells, specifically expanded in LNs, likely supported by increased CCL20 levels in the tumor tissue. At least in part, the presence of MCs could support this mechanism.

### 3.4 Antigen-experienced B cells able to expand into IgM^+^ memory cells and to differentiate into PBs *in vitro* were found from tumoral LN B cells

**Figure 4:**
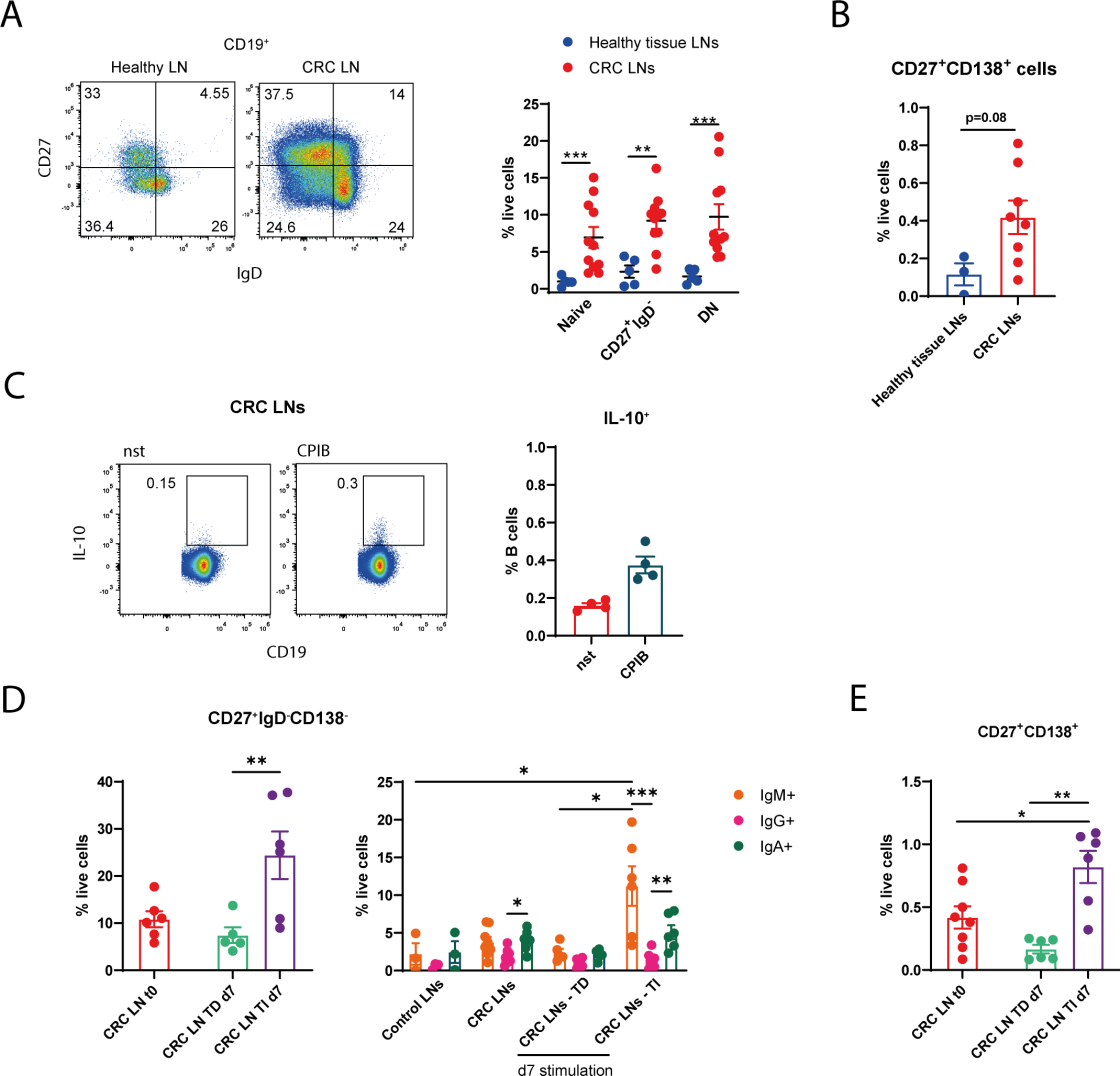
Antigen-experienced cells, able to expand into IgM^+^ memory cells and to differentiate into PBs *in vitro*, were found from tumoral LN B cells in human patients. Single cell suspensions of cancer or healthy tissue LNs were analyzed by flow cytometry. Percentages of **(A)** CD27^-^IgD^+^ (naive), CD27^+^IgD^-^ (antigen-experienced) or CD27^-^IgD^-^ double negative (DN) B cells, and of **(B)** IgD^-^CD27^+^CD138^+^ B cells are shown in the plots within total live cells. **(C)** Intracellular staining of IL-10^+^ CD19^+^ cells was performed after culturing LN cells in resting condition or upon stimulation with CpG (10 µg/ml), PMA (50 ng/ml), Ionomycin (1 µg/ml) and Brefeldin A (10 µg/ml) (CPIB) for 5h. Representative flow cytometry plots are shown. **(D)** Within total live cells, percentages of CD27^+^IgD^-^CD138^-^ cells were determined in CRC LN B cells at time 0 or 7 days after anti-CD40 (5 µg/ml) + IL-21 (50 ng/ml) T-dependent (TD) stimulation, or IL-2 (50 ng/ml) + CpG (3.2 µg/ml) T-independent (TI) stimulation. IgM-, IgG- or IgA–expressing CD27^+^IgD^-^CD138^-^ B cells are shown for both healthy tissue and CRC LNs at t0 or t7 upon TD or TI boost. **(E)** Percentages of IgD^-^CD27^+^CD138^+^ PBs/PCs were determined at t0 or d7 upon TD or TI boost within CRC LN live cells. Each symbol represents an individual patient. Means ± SEM are shown. Kruskal-Wallis analysis with uncorrected Dunn’s test was performed in **(A, D, E)** Mann-Whitney test has been used in **(B, C)** *p<0.05, **p<0.01, ***p<0.001.

The clinical relevance of B cells in CRC remains controversial, as tumor-infiltrating B cells have been associated with both pro- and anti-tumor functions (24). Furthermore, dissecting the roles of distinct functional B cell subsets may provide valuable insight into MC/B cell crosstalk in CRC. In the intestine, MCs provide co-stimulatory signals and cytokines that support B cell isotype switching and PC differentiation (3, 4) or, alternatively, promote regulatory B cell functions (25). Hence, we determined through flow cytometry the repartition of LN CD19^+^ B cells into IgD^+^CD27^-^ naïve B cells, IgD^-^CD27^+^ antigen-experienced B cells (which include activated/memory B cells and plasma blasts (PB)), and IgD^-^CD27^-^ double negative (DN) B cells. Regardless of the subset analyzed, all the aforementioned B cell populations were more abundant in CRC LNs with respect to healthy tissue LNs (**Figure 4A**; **Supplementary Figure 8A**). Within the IgD^-^CD27^+^ B cell population we determined the amount of plasma blasts/plasma cells (PBs/PCs) by utilizing the CD138 surface marker, obtaining a tendency towards increased PBs/PCs within CRC LNs (**Figure 4B**; **Supplementary Figure 8B**).

We further investigated two contrasting roles of B cells in the cancer setting: their regulatory, immune-tolerogenic function, and the expansion of memory B/PCs, which may contribute to anti-tumor immunity. To assess the regulatory function, we analyzed through intracellular staining IL-10^+^ cells within CD19^+^ CRC LN cells. Cells were cultured *ex-vivo* in resting (nst) condition or upon the CPIB boost (a stimulation composed of CpG + PMA + Ionomycin and Brefeldin A) which allows the evaluation IL-10-producing B cells (18). Under both conditions, a low percentage of IL-10^+^ B cells (around 0.2% and 0.3% of total B cells) was detected (**Figure 4C**). We then evaluated the percentage of germinal center or memory (IgD^-^CD27^+^CD138^-^) B cells, upon LN recollection from patients (t0) and 7 days after culturing cells in the presence of anti-CD40+IL-21 (T-dependent, TD) or CpG+IL-2 (T-independent, TI) stimulations. We obtained that IgD^-^CD27^+^CD138^-^ B cells expanded *in vitro* by utilizing the TI with respect of the TD boost (**Figure 4D**, left panel). Specific isotype signatures have been attributed to B cells in the TME of several cancer types (13, 26–29). Our analyses pointed out that most of this activated B cell population was composed of IgM^+^ and IgA^+^ cells, and only a minority was IgG^+^ (**Figure 4D** right panel; **Supplementary Figure 8B**). Furthermore, the IgM unswitched portion expanded upon TI stimulation. Additionally, 7 days post-stimulation, we observed an expansion of the PB/PC subset following TI boost (**Figure 4E**). Altogether, our data suggest that antigen-experienced B cells of CRC LNs are able, at least in part, to proliferate and to differentiate, possibly evoking an IgM anti-tumor B cell response. It is plausible that LN B cells are receptive to MC-derived support, which may facilitate their differentiation into PBs *in vivo*.

### 3.5 The interaction between MCs and CRC cells induces TNF-α release by MCs

**Figure 5:**
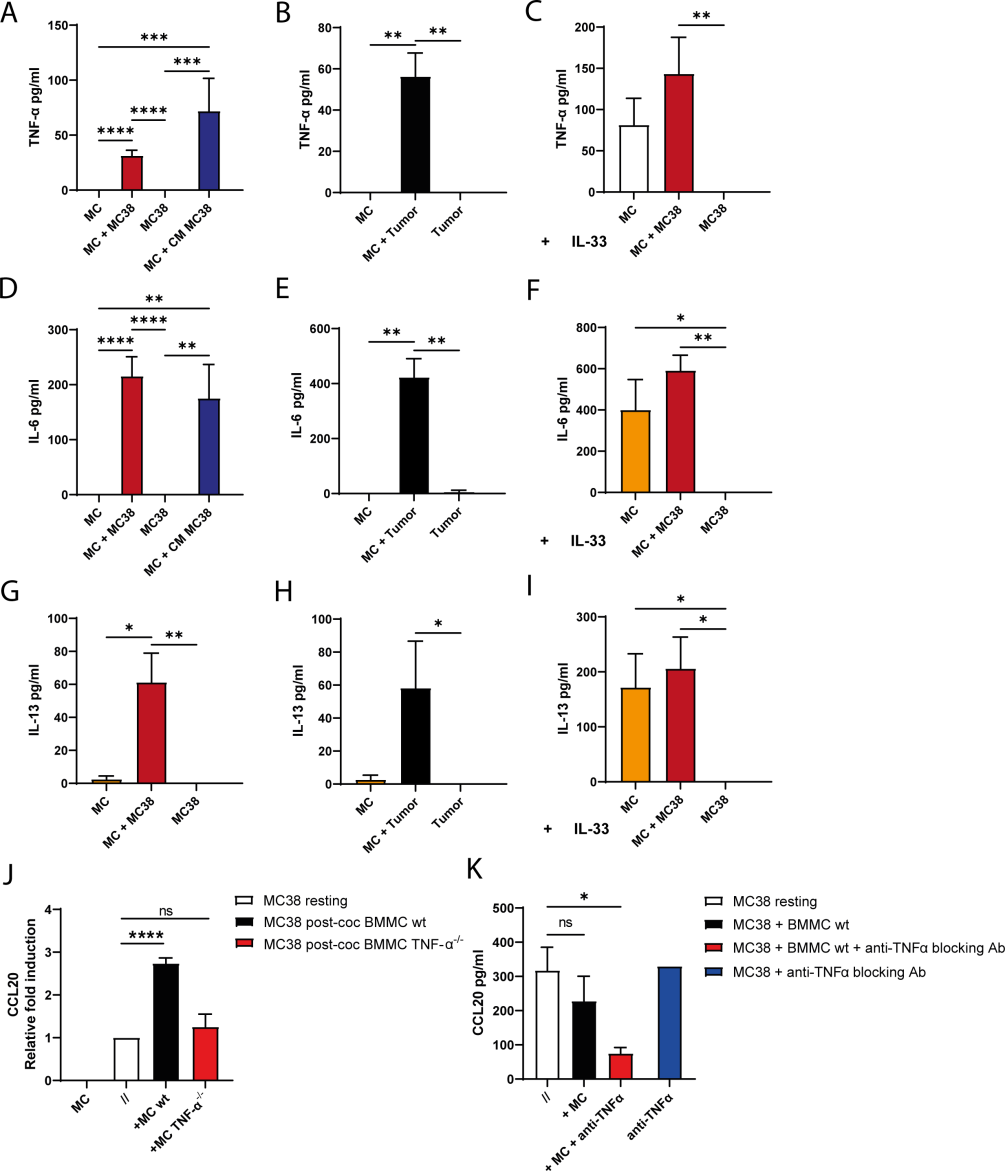
The activation of mouse MCs in CRC context fosters the increase of CCL20 production by cancer cells and is mediated by TNF-α. TNF-α **(A-C)**, IL-6 **(D-F)** and IL-13 **(G-I)** were measured by ELISA in 5h supernatants derived from: BMMCs (MC) and MC38 cell line cultured alone or in co-culture or MC cultured in the presence of 8h conditioned media (CM) derived from MC38 cells **(A, D, G)**; BMMCs cultured alone or with adherent cells derived from MC38 tumor mass grown in C57BL/6 mice (Tumor) **(B, E, H)**; 50 ng/ml of IL-33 was added to the culture medium **(C, F, I, J)**. Relative expression of mouse *Ccl20*, normalized to the housekeeping gene *G3pdh*, was determined in MC38 cells cultured for 5h alone (//) or in co-culture with BMMCs (+ MC). The same analysis has been performed by using wild-type (wt) or TNF-α^-/-^ BMMCs in parallel. **(K)** CCL20 concentration was evaluated through ELISA in the supernatants of MC38 cells cultured for 48h alone or with BMMCs in the presence or absence of 10 µg/ml TNF-α blocking mAb. Bar graphs represent averages +SEM of at least three independent experiments. *p<0.05, **p<0.01, ***p<0.001, ****p<0.0001 by Kruskal-Wallis analysis with uncorrected Dunn’s test **(C)**; ns=not statistically significant.

Our data show an accumulation of functional CCR6^+^ B cells in tumor LNs. MC levels also positively associate with serum CCL20 in patients (**Figure 3H**). To explore MC remodeling in proximity to CRC cells, we set up co-cultures between BMMCs and MC38 cells, cultured fresh or isolated from *in vivo* s.c. grown MC38 tumor mass (named Tumor). We assessed whether MC38 cells modulate MC-derived soluble factors in co-culture, with or without IL-33, focusing on TNF-α, IL-6, and IL-13, key pro-inflammatory mediators in cancer. All cytokines analyzed were undetectable when MCs and MC38 or Tumor cells were cultured separately, while they emerged in the coculture supernatant (**Figures 5A, B, D, E, G, H**), or when MCs were stimulated by IL-33 (**Figures 5C, F, I**). We focused on TNF-α release, given its debated association with CRC progression (30, 31) and its impact on tumoral organoid viability and phenotype, as previously shown by our group (21). Confirmation that TNF-α cytokine derives from MCs is provided by the establishment of the co-culture system with TNF-α knock-out (TNF-α^-/-^) MCs where no TNF-α is detectable in any condition (**Supplementary Figure 10A**). Moreover the CM derived from MC38 is able by itself to induce both TNF-α and IL-6 release by MCs (**Figures 5A, D**), this suggests that direct MC–MC38 contact is not required, and that MC38-derived soluble factors at steady state are sufficient to trigger MC release of pro-inflammatory mediators. MC38-derived SCF, a potent MC activator in cancer (15, 16), emerged as a candidate for the observed effect. We confirmed its release by both resting and IL-33–stimulated MC38 cells, and its likely consumption by MCs in co-culture (**Supplementary Figures 10B, C**).

### 3.6 The activation of MCs in CRC context fosters the increase of CCL20 production by cancer cells and is mediated by TNF-α

We proceeded testing whether MC-CRC cells interaction could modulate CCL20 expression in MC38 cells, supposing that cancer cells are a major source of CCL20 in the TME. While resting BMMCs did not express any CCL20 transcript through qPCR analysis (nor after contact with MC38 cells, **Supplementary Figure 11**), in MC38 cells CCL20 mRNA expression significantly increased when cells were cultured with BMMCs (**Figure 5J**). In panel A we showed that MC38 cells activate MCs to release TNF-α. In several inflammatory and cancer contexts, TNF-α has been implicated in the induction of CCL20 (32, 33), and as a confirmation, we proved a dose-dependent increase of CCL20 release by MC38 cells upon stimulation with recombinant TNF-α (**Supplementary Figure 12**). We then investigated whether CCL20 mRNA modulation in MC38 cells was impaired if co-cultured with TNF-α^-/-^ BMMCs. The analysis showed that in the presence of TNF-α^-/-^ BMMCs CCL20 was not upregulated on MC38 cells, indicating that MC-derived TNF-α can reasonably explain the upregulation of CCL20 by CRC cells (**Figure 5J**). In order to confirm the role of TNF-α released by MCs in CCL20 production, we quantified through ELISA the CCL20 chemokine in the MC38/MC co-culture system by also utilizing a TNF-α blocking antibody. By blocking soluble TNF-α, we attained significant reduction of CCL20 production by cancer cells in contact with BMMCs compared to the resting MC38 cell condition (**Figure 5K**). Using transcriptomic data of CRC patients from the TCGA, we performed correlation analysis between CCL20 and TNF-α obtaining moderate positive correlation (**Supplementary Figure 13A**). We also found moderate positive correlation between CCL20 and CCR6 (**Supplementary Figure 13B**) and between CCL20 and IL-33 (**Supplementary Figure 13C**). Finally, we used immunofluorescence analysis to validate TNF-α expression in MCs infiltrating human CRC biopsies. No colocalization between TNF-α signal and Tryptase^+^ MCs within healthy colon tissue was observed while, association between the two markers was detected in MCs infiltrating CRC tissue (**Supplementary Figure 14**). CCL20 signal appeared enriched in CRC tissues (**Supplementary Figure 14**), confirming histological analysis shown in **Figure 3G**.

Altogether these findings support previous evidence identifying MCs as a major source of TNF-α in CRC (21, 34), and reinforce the central hypothesis of this study, highlighting a previously unrecognized role of MC-derived TNF-α in driving CCL20 production in the CRC microenvironment. **Supplementary Figure 15** provides a comprehensive model summarizing our findings and outlining the key mechanisms involved in the process.

## 4 Discussion

Inflammatory mediators and chemokines are essential modulators of the TME that impact tumor progression in multiple ways. Emerging evidences emphasize the primary importance of the CCL20 chemokine in shaping CRC TME (35). CCL20 is both a diagnostic marker and therapeutic target in CRC, where it plays a dual role by either promoting or suppressing tumor progression. Frequently upregulated in CRC, CCL20 contributes to tumor development by recruiting CCR6^+^ immunosuppressive cells such as regulatory T cells (Tregs) and myeloid-derived suppressor cells (MDSCs), and by promoting processes like epithelial–mesenchymal transition (EMT), angiogenesis, and metastasis (36–38). Conversely, CCL20 can enhance anti-tumor immunity by attracting CCR6^+^ effector T cells and dendritic cells, supporting immune surveillance (39, 40). This duality underscores the complexity of targeting CCL20 therapeutically, as its inhibition may reduce tumor-promoting inflammation but also compromise beneficial immune responses. In an effort to unravel the complexity of CCL20’s role in CRC, our study reveals a previously unrecognized anti-tumorigenic property. We propose that CCL20 contributes to the recruitment of CCR6^+^ B cells within LNs in the TME. Presence of LNs and tertiary lymphoid structures (TLS) localized predominantly at the tumor edges, have been observed across various cancers, including CRC. Generally, a favorable prognosis has been associated to the occurrence of these structures - especially when localized intra-tumor - believed to represent a local anti-tumor immune response (41–46). CCL20 and other chemokines increase the capacity to attract lymphocytes, fostering formation of TLS (46), thus understanding the factors that promote lymphoid aggregates formation becomes crucial to develop novel approaches to treat cancer, especially in early stage. In patients’ CRC biopsies, we observed a preferential accumulation of CCL20 in the peritumoral stroma which may enhance immune cell trafficking at the tumor margin: this area is therefore likely to facilitate lymphocyte recruitment and infiltration more effectively than the inner tumor core, where high cellular density and limited stromal space may restrict chemokine accumulation and diffusion (**Figure 3G**). This work suggests that MCs, activated by the CRC TME (9, 21, 23, 34) (**Figure 5**), could indirectly facilitate the recruitment of B lymphocytes in the TME (**Figures 2**, **3**). SCF and IL-33 are highly expressed in CRC, promoting MC accumulation and activation, and driving their production of inflammatory mediators such as TNF-α and IL-6 (15, 16, 47). IL-33 plays a significant role in tumor progression, particularly in liver metastasis. Evidence from the MC38 tumor model suggests that IL-33 promotes angiogenesis (48). TNF-α, similarly to CCL20, exhibits a paradoxical role in CRC, acting as both a tumor promoter and suppressor depending on the context (49). On the one hand, TNF-α contributes to tumor progression by fostering a pro-inflammatory microenvironment, promoting angiogenesis, and supporting the survival and proliferation of malignant cells. On the other hand, TNF-α has been shown to exert anti-tumor effects by inducing cancer cell apoptosis, enhancing immune surveillance, and activating cytotoxic immune responses. This duality reflects the complex interplay between inflammation and immunity in CRC and underscores the challenge of targeting TNF-α therapeutically without disrupting its beneficial roles. Our findings suggest that manipulating TNF-α-derived MCs in early phases of CRC context could enable patients to benefit from increased levels of CCL20, a mediator that can activate immune surveillance. Further elucidation is required to define its contribution to B cell–driven immune control mechanisms in tumor advancement. In the human context we are unable to precisely determine the role of TNF-α released by human MCs in the cancer tissue and its direct impact in the production of CCL20 and recruitment of B lymphocytes, due to the conceptual impossibility of studying in patients the impact of the absence of MCs during tumor development. In Kit^W-sh^ dLNs, B cells, albeit accumulated (**Supplementary Figure 4**), expressed lower levels of CCR6 receptor compared to the BMMC rec-Kit^W-sh^ dLNs (**Figure 2B**). MC-mediated modulation of B cell surface molecules has been previously demonstrated for CD86 and MHC-II (4, 20). We showed here that MCs, activated in an IL-33 dependent-context, induce upregulation of CCR6 on B cells, even not requiring direct cell-to-cell contact (**Figure 2A**). More interestingly, in Kit^W-sh^ MC-lacking mouse we determined that in the absence of MC-invading the s.c. tumor, infiltrating B cells were decreased along with diminished CCL20 positivity (**Figures 2D, E**). All these data suggest a role of MCs in regulating the recruitment of B cells through the CCR6/CCL20 axis. In support to our hypothesis, a positive correlation between MCs and B cells among immune cells in human CRC and between MCs and CCL20 serum levels in patients were obtained (**Figures 3C, H**). It is plausible that tumor-activated MCs create a milieu that enhances the sensitivity of a B cell to a CCL20 gradient. Using multi-omics and single-cell sequencing, Zhang and colleagues showed functional heterogeneity among B-cell subsets in CR, including antibody-producing cells and inflammation-regulating populations, which could explain their contrasting roles (8). We demonstrated for the first time (to the best of our knowledge) through *ex-vivo* experiments that B cells localized within CRC LNs express higher levels of CCR6, compared to B cells interspersed in the tissue, and are able to differentiate into PB/PC, rather than showing a regulatory pro-tumor IL-10 producing phenotype (**Figures 3**, **4**). It has been shown that in CRC high infiltration of CD20^+^ B cells and CD138^+^ plasma cells is associated with improved patient survival (11, 50). CCL20/CCR6-driven accumulation of B cells, potentiated by TNF-α released by activated MCs, could therefore represent a positive axis in driving an anti-tumor response. The contradictory role of MC–derived TNF could be reconciled by a model in which early-stage release of the mediator could be beneficial by promoting B cell–mediated immune surveillance, whereas in later stages it could become detrimental by enhancing tumor aggressiveness. Another intriguing aspect of MC involvement in CRC development is represented by contradictory evidences on patient prognosis related to high/low density of infiltrating MCs (51–55). It is reasonable to assume the effects of MCs in tumorigenesis and cancer development to be much more related to their activation - modulated by the specific cues from the TME - than to their relative density. This concept has been already proposed by other authors who, using spatial transcriptomic analysis, observed lower MC density in tumor than in normal colon (in line with data shown in **Figure 3B**) along with substantial change in activated phenotype compared to the physiological condition (23). In their single cell RNA-seq analysis, Xie, Z. et colleagues revealed TNF-α signature through NF-kB signaling the most enriched in activated MCs. Accordingly, our confocal staining showed colocalization of Tryptase and TNF-α signals in MCs from tumor tissue (**Supplementary Figure 14**). Integrating immune cell density, activation status, and inflammatory markers will ultimately enable risk-based patient stratification, advancing precision medicine.

## Data Availability

The raw data supporting the conclusions of this article will be made available by the authors, without undue reservation.
