# Supplementary material for: Mast cell release of TNF-α indirectly contributes to recalling B cells in the colorectal cancer milieu through the CCL20/CCR6 axis
Source: Front Immunol. 2026 Apr 21;17:1725902. doi: 10.3389/fimmu.2026.1725902 (PMC13139354; doi:10.3389/fimmu.2026.1725902)
Supplement: Supplementary file 1 [file DataSheet1.pdf]

Supplementary Material

Mast cell release of TNF-α indirectly contributes to recalling B cells in the colorectal cancer milieu through the CCL20/CCR6 axis

Correspondence: Viviana Valeri; viviana.valeri@uniud.it

Supplementary Table 1. List of antibodies and reagents used for flow cytometry analyses

Antibody or reagent	Fluorophore	Manufacturer	Catalogue number
Anti-human CD45	FITC	Sony	2120030
Anti-human CD45	PE-AF610	Invitrogen	MHCD4522
Anti-human CD19	APC	Sony	2111060
Anti-human CD19	ef450	Invitrogen	48-0198-42
Anti-human CD20	BV510	Invitrogen	302339
Anti-human CD21	SB600	Invitrogen	63-0219-42
Anti-human CD24	PE-Dazzle594	Biolegend	311133
Anti-human CD27	PE-Cy7	Sony	2114190
Anti-human CD38	BV421	Sony	2117630
Anti-human IgM	PerCP-Cy5.5	Biolegend	314511/2
Anti-human IgD	SB702	Invitrogen	67-9868-42
Anti-human CD138	PE-Dazzle594	Sony	2382650
Anti-human IgG	BV421	Biolegend	2653515
Anti-human IgA	PE	Miltenyi	130-113-476
Anti-human CD196 (CCR6)	PE	Sony	2367050
Anti-human FcERI	SB600	Invitrogen	63-5899-42

Anti-human CD117 (cKit)	APC	Invitrogen	17-1178-41
Anti-human CD4	ef506	Invitrogen	69-0049-42
Anti-mouse CD19	FITC	eBioscience	11-0193
Anti-mouse CD19	APC	BioLegend	115512
Anti-mouse CD19	APC-Fire	BioLegend	115557
Anti-mouse CD196 (CCR6)	PE	Biolegend	129803
Anti-mouse CD4	FITC	BDPharmingen	553047
Live/Dead	Lime	Invitrogen	L34990 A
Live/Dead	eFluor780	Invitrogen	65-0865-14
Live/Dead	Violet	Invitrogen	L34958
CD16/CD32 mAb (Fc-block)		Invitrogen	16-0161-82
Super bright buffer		Invitrogen	SB-4401-75

Supplementary Table 2. List of primers used for qPCR experiments.

**Target gene**	**Forward primer**	**Reverse primer**
G3PDH *mouse*	TCAACAGCAACTCCCACTCTTCCA	ACCCTGTTGCTGTAGCCGTATTCA
CXCL12 *mouse*	GGTAGCTCAGGCTGACTGGT	TCCTCTTGCTGTCCAGCTCT
CXCL13 *mouse*	CATAGATCGGATTCAAGTTACGCC	TCTTGGTCCAGATCACAACTTCA
CXCL19 *mouse*	CTGTGGCCTGCCTCAGATTA	GTGTGGTGAACACAACAGCA
CCL20 *mouse*	TGCTATCATCTTTCACACGAAGAA	TCATTTCCTCCTTGGGCTGT
CCL21 *mouse*	ATCCCGGCAATCCTGTTCTC	TTCTCTTGGGCTGT
Beta2-microglobulin *human*	CCAGCAGAGAATGGAAAGTC	GATGCTGCTTACATGTCTCG

CCL20 *human*	CTGCTTTGATGTCAGTGCTGC	TCACCCAAGTCTGTTTTGG

Supplementary Table 3. List of Antibodies and reagents used for human IF experiments.

**Antibody and reagents**	**Catalog**	**Manufacturer**
α-Tryptase mouse	MA5-11711	Invitrogen
α-TNFα rabbit	ab66579	Abcam
α-CCL20 goat	PA5-47517	Invitrogen
donkey α-mouse AlexaFluor®488	715-545-150	Jackson ImmunoResearch
donkey α-rabbit AlexaFluor®555	A31572	Invitrogen
donkey α-goat AlexaFluor®647	A-21447	Invitrogen

Supplementary Table 4. CRC patients information.

Characteristics	Value
Age, years (mean ± SD)	74.7 ± 10
Sex (%)	55.6 % F; 44.4 % M

Cell media composition:

Complete DMEM. DMEM supplemented with 10% FBS, 2 mM L-glutamine, 100 U/ml penicillin and 100 μg/ml streptomycin.

Complete RPMI. RPMI supplemented with 10% FBS, 20 mM Hepes, 2 mM L-glutamine, 1 mM sodium pyruvate, 100 U/ml penicillin, 100 μg/ml streptomycin and 50 mM β-mercaptoethanol.

Supplementary figure 1

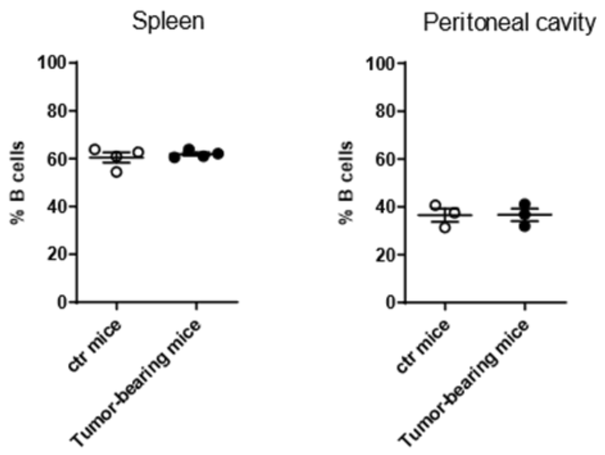

Suppl. figure 1: B cell frequencies in the spleen and peritoneal cavity remained unchanged in tumor-bearing mice compared to controls. Percentages of $CD19^+$ B cells were determined by flow cytometry from splenocytes (on the left) and peritoneal lavages (on the right) of control (ctr) and s.c. MC38-tumor bearing mice. No statistically significant differences were observed using the Mann–Whitney test.

Supplementary figure 2

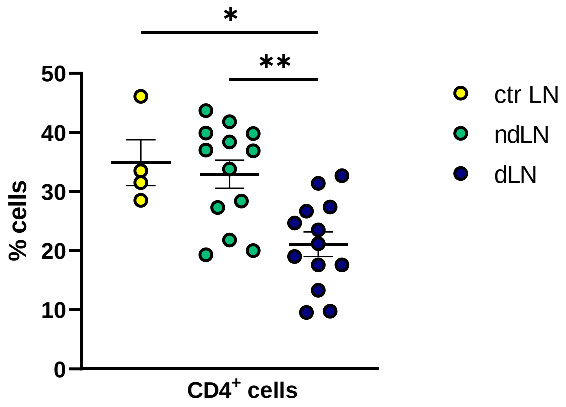

Suppl. figure 2: $CD4^+$ T cells are decreased in tumor-draining LNs of MC38 tumor mice. Percentages of $CD4^+$ T cells were analyzed by flow cytometry in cell suspensions obtained from control (ctr LN) or draining (dLN) and non-draining (ndLN) LNs of MC38 tumor-bearing mice. Statistical significance was assessed using the Kruskal–Wallis test followed by uncorrected Dunn's post hoc test. *p<0.05, **p<0.01.

Supplementary figure 3

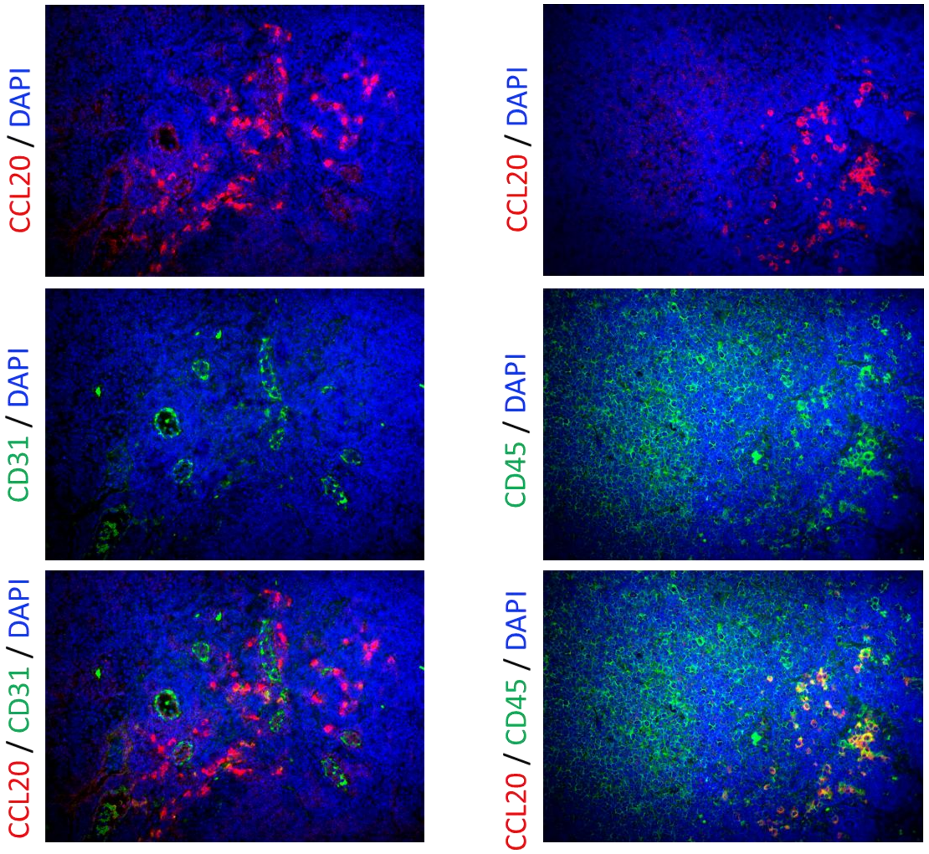

Suppl. figure 3: CD31^{+} endothelial cells and CD45^{+} immune cells exhibited detectable co-staining with CCL20 in tumor-draining LNs of MC38 tumor mice. Representative double-immunofluorescence stainings for CCL20 and CD31 (left) or CD45 (right), together with DAPI. Magnification: 20x.

Supplementary figure 4

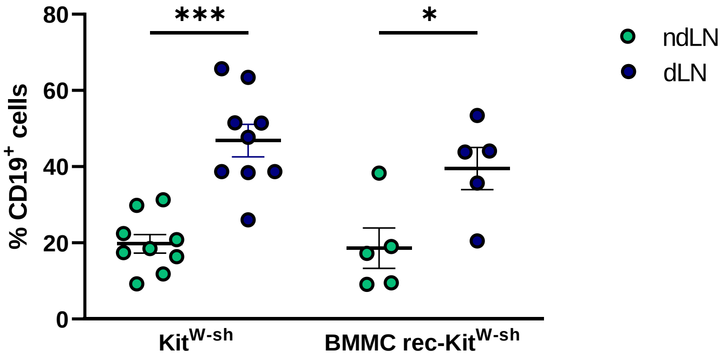

Suppl. figure 4: B cell frequencies in tumor-bearing mice LNs. Percentages of CD19^{+} cells were analyzed by flow cytometry in cell suspensions obtained from not-draining lymph nodes (ndLNs) and draining LNs (dLNs) of Kit$^{W\text{-}sh}$ and BMMC reconstituted (rec) Kit$^{W\text{-}sh}$ MC38 tumor-bearing mice. Kruskal-Wallis analysis with uncorrected Dunn's test was performed. *p<0.05, ***p<0.001.

Supplementary figure 5

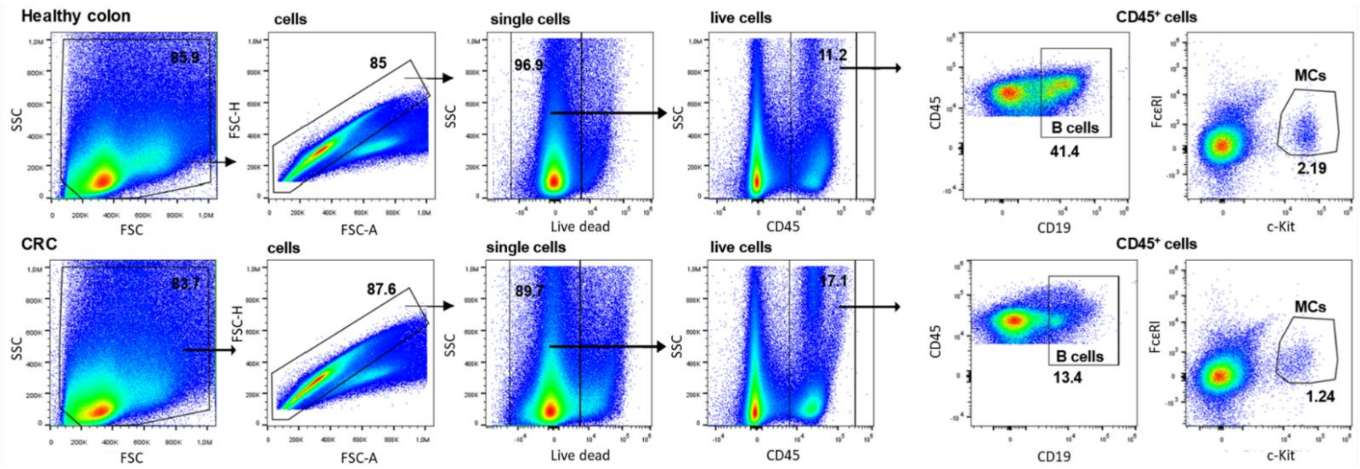

Suppl. figure 5: Gating strategy approach used on cell suspensions prepared from freshly processed healthy and tumor colon biopsies. Cells were initially selected based on morphological parameters, and doublets were excluded from the analysis. Live single cells were then gated, followed by the identification of $CD45^+$ tissue-infiltrating immune cells. Specific subsets of interest, including $CD19^+$ B cells and $Fc\varepsilon RI^+c\text{-}Kit^+$ MCs, were subsequently isolated by gating.

Supplementary figure 6

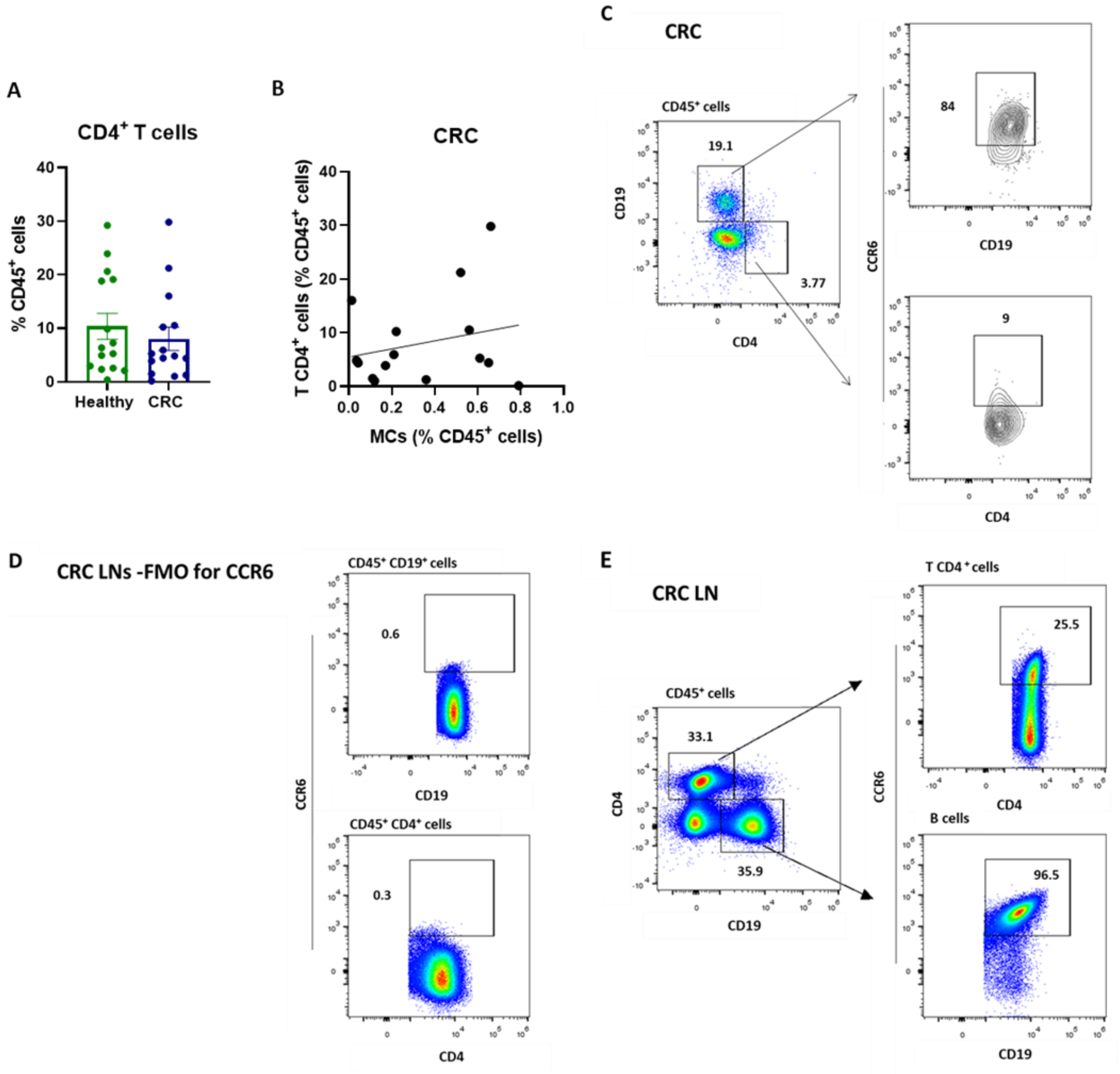

Suppl. figure 6: A small amount of CCR6$^+$ CD4$^+$ T cells infiltrate CRC samples. (A) CD4$^+$ T cells were determined through flow cytometry analysis performed on digested cell suspension of both healthy colon and CRC tissue and are shown as percentages of CD45$^+$ cells. **(B)** Spearman correlation analysis has been conducted between CD4$^+$ T cells and MCs from CRC tissue; r= 0.08571 and p= 0.7630. **(C)** CCR6 expression on both CD19$^+$ B cells and CD4$^+$ T cells has been determined from digested colon. FMO **(D)** and CCR6 **(E)** flow cytometry staining for both CD19$^+$ and CD4$^+$ cells is shown from CRC LNs.

Supplementary figure 7

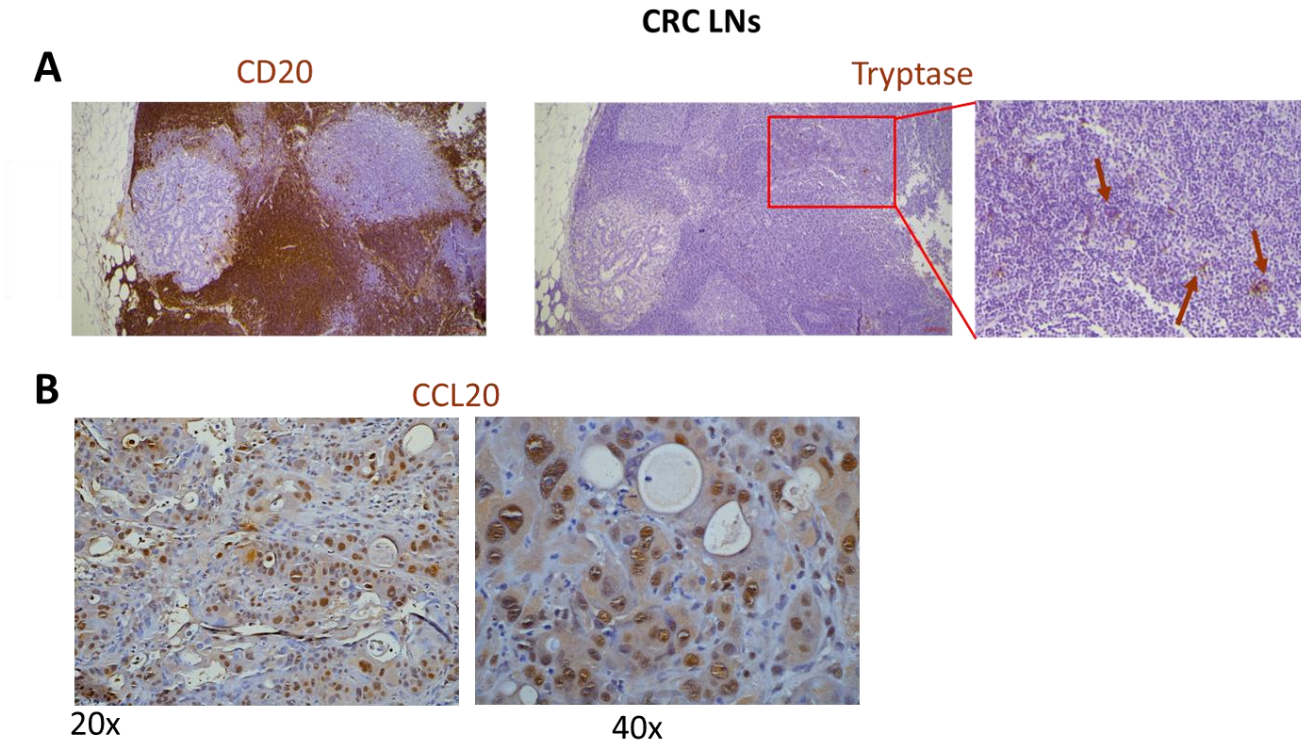

Suppl. figure 7: Histological analyses of human CRC lymph nodes. (A) Representative immunohistochemical staining of B cells (CD20, left) and MCs (Tryptase, right) in LNs infiltrating the tumor. A higher magnification image of the Tryptase staining is also shown. **(B)** Representative immunohistochemical staining of $CCL20^+$ cells in tumor associated LNs. Two magnifications are presented.

Supplementary figure 8

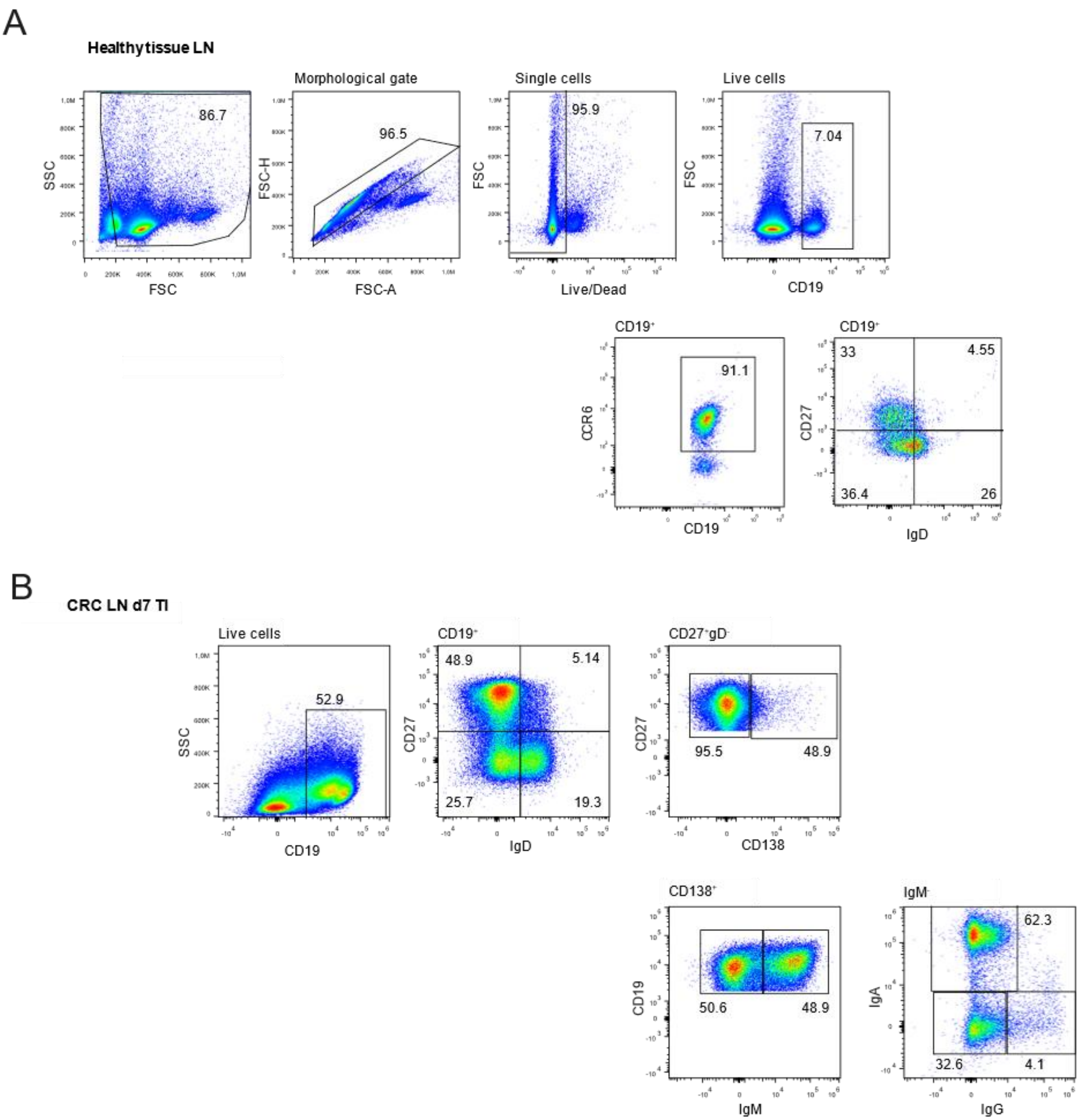

Suppl. figure 8: Flow cytometry gating strategy for the analysis of B cells from intestinal LNs. Cells were initially selected based on morphological parameters, and doublets were excluded from the analysis. Live single cells were then gated, followed by the identification of CD19^{+} lymph node B cells. **(A)** B cells were analyzed for CCR6 expression or for the alternative expression of IgD and CD27, which allows the distinction between naïve B cells (IgD^{+}CD27$^{–}$), antigen-experienced B cells (IgD$^{–}$CD27^{+}) or double-negative B cells (IgD$^{–}$CD27^{-}). **(B)** Within the antigen-experienced population, CD138 expression was used to further differentiate between germinal center/memory B cells (CD138$^{–}$)

and plasma blasts/plasma cells (CD138⁺). Germinal center and memory B cells were subsequently characterized based on their expression of IgM, IgG, and IgA.

Supplementary figure 9

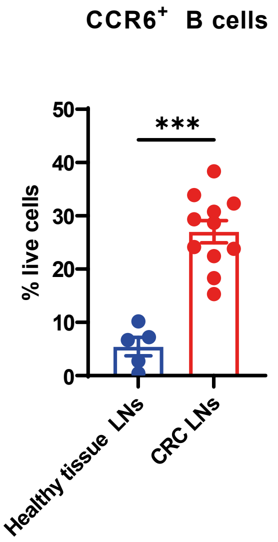

Suppl. figure 9: Accumulation of CCR6^{+} B cells in CRC LNs. Percentages of CCR6^{+} B cells were determined in healthy and tumor LNs by flow cytometry.

Supplementary figure 10

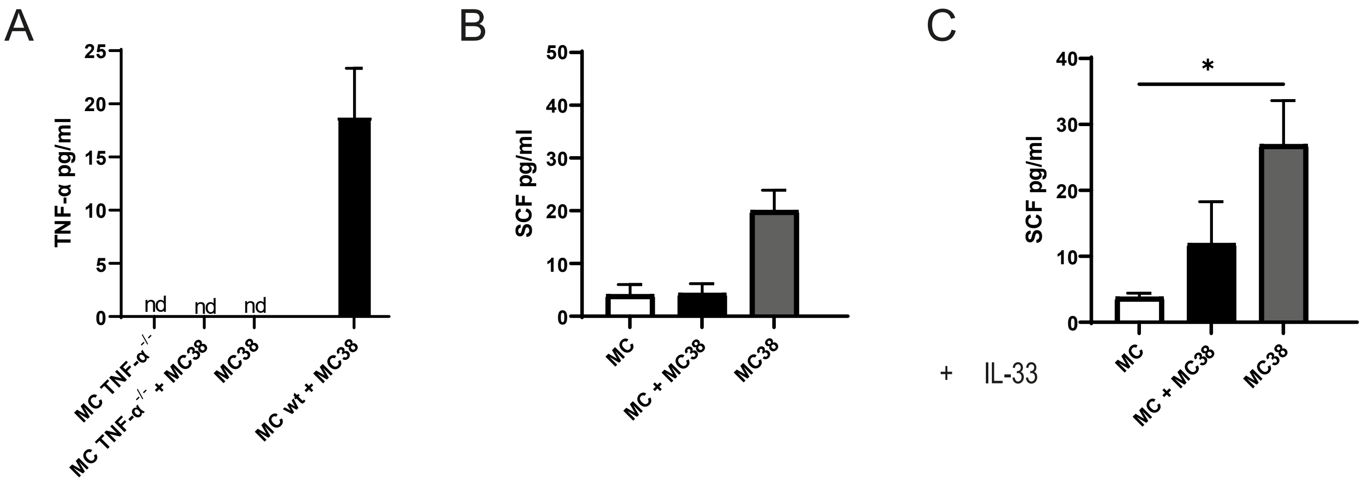

Suppl. figure 10: In the interaction between BMMCs and MC38 cells, the release of TNF-α by BMMCs may be driven by SCF secretion from the cancer cells. (A) TNF-α levels were quantified in the BMMC/MC38 co-culture system using ELISA, employing either wild-type or TNF-α$^{-/-}$ BMMCs as indicated. SCF production was further assessed in the absence **(B)** or presence **(C)** of 50 ng/ml IL-

33. Statistical significance was assessed using the Kruskal–Wallis test followed by uncorrected Dunn's post hoc test. *p<0.05. nd=not determined.

Supplementary figure 11

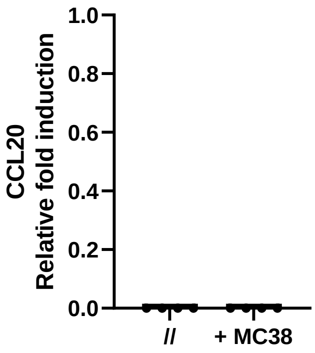

Supplementary figure 11: Relative expression of mouse *Ccl20*, normalized to the housekeeping gene *G3pdh*, was determined in BMMCs cultured for 5h alone (//) or in co-culture with MC38 cells (+ MC38).

Supplementary figure 12

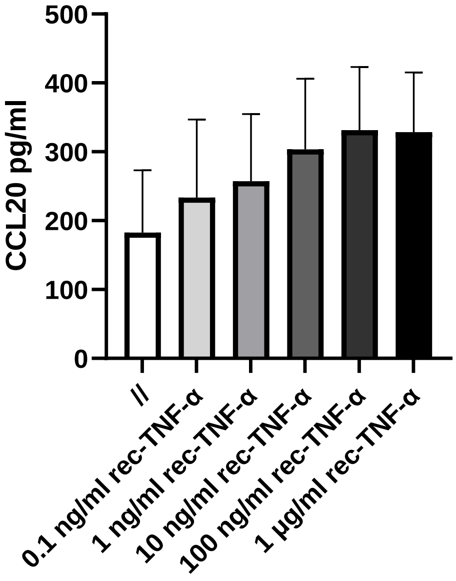

Supplementary figure 12: CCL20 concentration was evaluated through ELISA in the supernatants of MC38 cells cultured for 24h alone (//) or in the presence of increasing concentrations of recombinant mouse TNF-α. Bar graphs represent averages +SEM of three independent experiments.

Supplementary figure 13

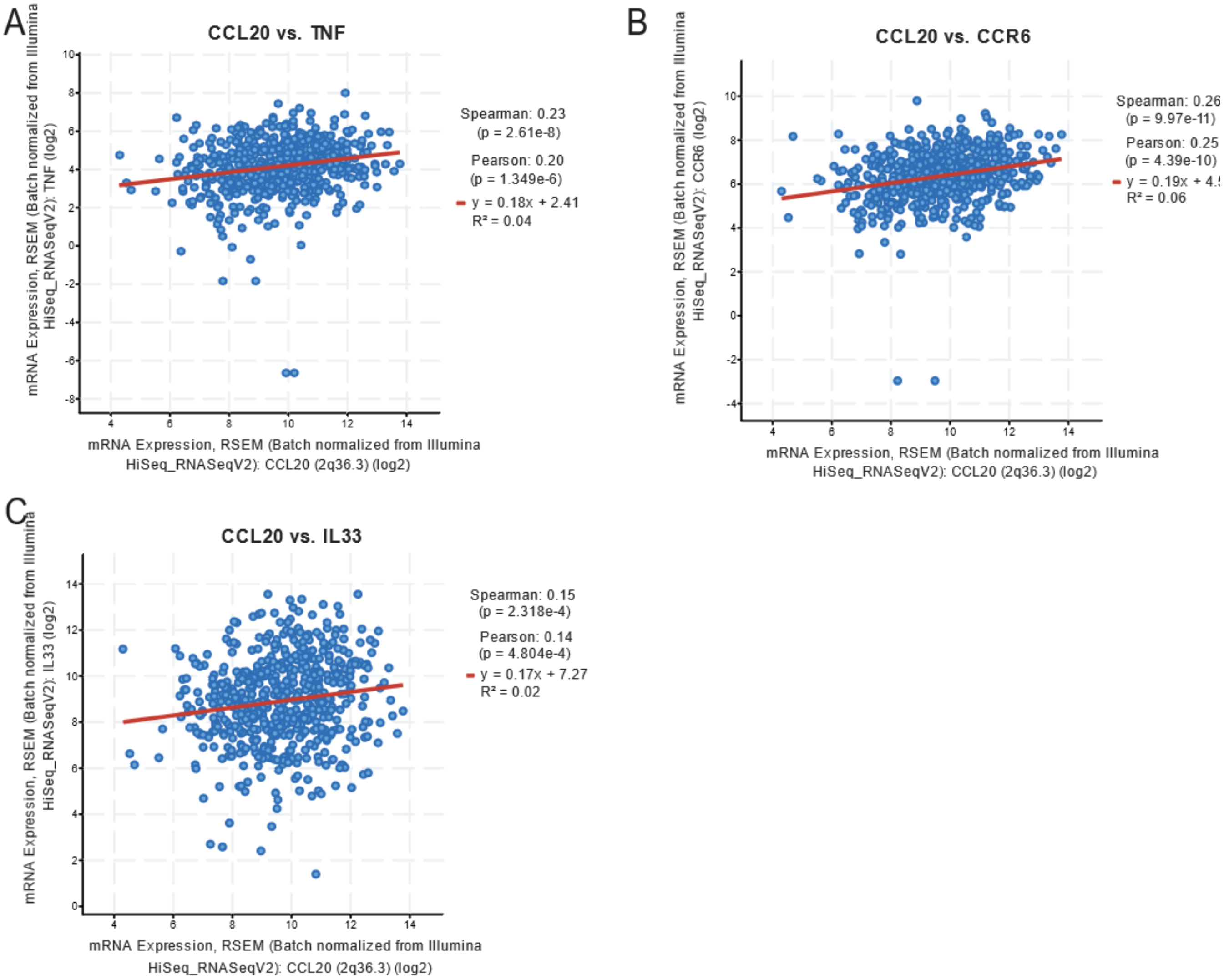

Suppl. figure 13: Correlation analyses of selected markers were performed using transcriptomic data from CRC patients in the TCGA dataset (Illumina HiSeq platform). (A) CCL20 versus TNF-α, **(B)** CCL20 versus CCR6, and **(C)** CCL20 versus IL-33 correlation plots are shown. Both Spearman and Pearson correlation p-values and coefficients are reported in the graphs.

Supplementary figure 14

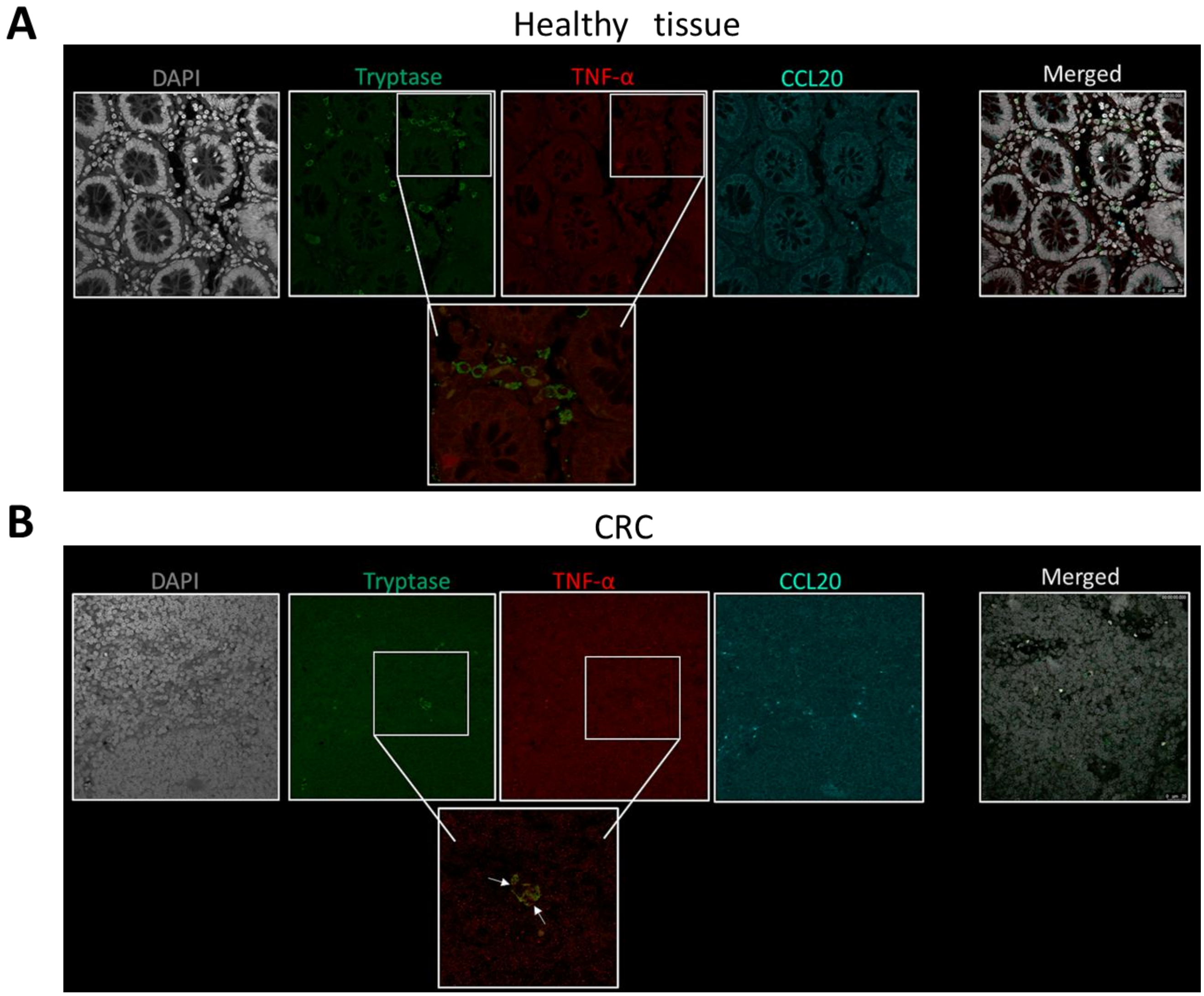

Suppl. figure 14: TNF-α⁺ MCs are localized within CRC biopses. Representative immunofluorescence analysis for Tryptase in green (MCs), TNF-α in red and CCL20 in cyan from paraffin embedded samples of both healthy or tumor colon samples from CRC patients. Paraffinized tissue sections were dewaxed at 60°C for 1h and rehydrated with xylene (30 min) followed by alcohols scale (100% > 95% > 70% > 5029% > 35% > H2O) (2 min each). After rehydration, antigen unmasking has been performed with 20' boiling in Antigen Retrieval solution (pH6 Na citrate 0,5% Tween-20) followed by 10 min permeabilization with Tryton 1% PBS. Slides were blocked >1h with BSA 5% PBS and incubated with primary antibody 2h RT followed by 1h incubation with secondary antibody, both reactions in BSA 1% PBS. Washing steps were performed using PBS 1x. VECTASHIELD HardSet™ (Fisher Scientific, Cat. No. NC9029228) was used as mounting medium. For double and triple staining, sequential rounds of single-marker immunostaining (primary + secondary Ab) were performed. Leica TCS SP8 Confocal Microscope was used to acquire IF images.

Supplementary figure 15

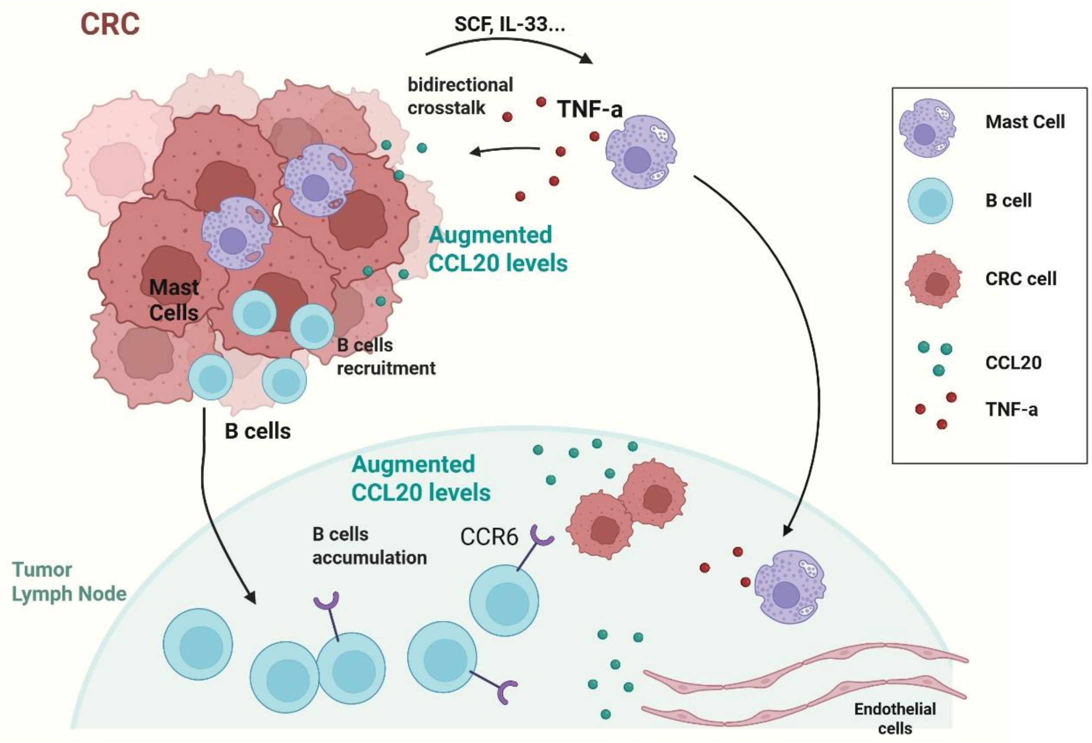

Suppl. figure 15: Comprehensive model summarizing our findings and the key mechanisms involved. In CRC, a bidirectional crosstalk is established between MCs and cancer cells. Tumor-derived signals such as SCF and IL-33 promote MC activation and the release of inflammatory mediators, including TNF-α. TNF-α, in turn, sustains the production of the CCL20 chemokine. This chemokine acts as a key chemotactic factor driving the recruitment of B cells into the tumor and promoting the accumulation of CCR6^{hi} B cells within tumor-associated lymphoid structures, particularly when cancer cells can be found within LNs in patients. In mice, endothelial and additional immune cells can also contribute to the increased production of the CCL20 in LNs proximal to the subcutaneous tumor model. Created with Biorender.com.

frontiers
